# Uncoupling Metastasis and Epithelial‐to‐Mesenchymal Transition in sgP19/kRAS‐Driven Spontaneous Metastatic Liver Tumor Model

**DOI:** 10.1002/advs.202514198

**Published:** 2025-12-07

**Authors:** Jingwen Wang, Zijing Xu, Lei Xu, Lishan Wang, Jiahao Geng, Xue Wang, Meng Xu, Daphne Superville, Melissa Reeves, Matthias Evert, Diego F. Calvisi, Xin Chen, Xinhua Song

**Affiliations:** ^1^ School of Traditional Chinese Medicine Laboratory for Clinical Medicine Capital Medical University Beijing 100069 China; ^2^ Department of Gastroenterology Nanjing Drum Tower Hospital The Affiliated Hospital of Nanjing University Medical School Nanjing Jiangsu Province 210008 P. R. China; ^3^ Department of Gastroenterology Nanjing Drum Tower Hospital Clinical College of Nanjing University of Chinese Medicine Nanjing Jiangsu Province 210008 P. R. China; ^4^ Cancer Biology Program University of Hawai'i Cancer Center University of Hawai'i Honolulu Hawaii HI 96813 USA; ^5^ Department of General Surgery The Second Affiliated Hospital of Xi'an Jiaotong University Xian Shaanxi Province 710004 P. R. China; ^6^ Department of Cell and Tissue Biology UCSF San Francisco CA CA 94143 USA; ^7^ Huntsman Cancer Institute University of Utah Salt Lake City UT UT 84112 USA; ^8^ Department of Pathology University of Utah Salt Lake City UT Utah 84112 USA; ^9^ Institute of Pathology University of Regensburg 93053 Regensburg Germany; ^10^ Department of Medicine, Surgery, and Pharmacy University of Sassari Sassari 07100 Italy

**Keywords:** epithelial‐to‐mesenchymal transition, intrahepatic cholangiocarcinoma, metastasis, TGFβ, ZEB1

## Abstract

Epithelial‐to‐mesenchymal transition (EMT) is an early event during tumor metastasis. Here, the relevance of EMT in liver carcinogenesis and metastasis is sought to be determined in a murine mixed typical intrahepatic cholangiocarcinoma (iCCA)/sarcomatoid iCCA liver tumor model using CRISPR/Cas9‐based gene deletion of p19 (*sgP19)* in combination with transposon‐based expression of the activated form of pCaggs‐kRAS^G12D^ (*kRAS)* in the mouse liver (*sgP19/kRAS* mixed model). It discovered that metastasis in the lymph node, lung, or kidney occurred in the *sgP19/kRAS* mixed model. Both typical iCCA tumor cells with epithelial features and sarcomatoid tumor cells with mesenchymal features could be detected in this model. Lineage tracing technology is applied to confirm the metastasis induced in the *sgP19/kRAS* model. Subsequently, the gain of expression of mesenchymal marker vimentin in tumor cells revealed the induction of EMT in the *sgP19/kRAS* model, and it is induced by activating the TGFβ/ZEB1 signaling pathway. Altogether, the study suggests that TGFβ/ZEB1 mediates the induction of EMT in iCCA, while targeting EMT failed to inhibit iCCA development or tumor metastasis, disputing the claims that EMT is a major molecular event leading to tumor metastasis.

## Graphical abstract

The article first reported that a murine mixed typical intrahepatic cholangiocarcinoma (iCCA)/sarcomatoid iCCA liver tumor model with 100% incidence of spontaneous extrahepatic metastasis and confirmed the occurrence of in mouse iCCA. Most importantly, EMT induced by the TGF‐β/ZEB1 axis does not influence tumor development or distant metastasis in this model.

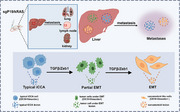

## 1 Introduction

Intrahepatic cholangiocarcinoma (iCCA) is an aggressive tumor with a poor prognosis due to its late clinical presentation and the lack of effective non‐surgical therapies.^[^ ^1^, ^2^ ^]^ Surgical resection is the only curative treatment. Unfortunately, most of the patients are not eligible for curative surgery owing to the presence of metastasis at the time of diagnosis.^[^ ^3^ ^]^ Meanwhile, the success of surgical resection is heavily threatened by the high rates of recurrence, resulting in a five‐year overall survival rate lower than 10%.^[^ ^3^ ^]^ Therefore, it is urgent to understand the steps leading to metastasis in patients with iCCA, which might help identify novel therapeutic strategies for this disease.

Considerable evidence demonstrates that lymph node metastasis has been one of the prominent predictors of malignant traits and poor outcomes following surgery for iCCA.^[^ ^4^ ^]^ The lung was considered the most common site of iCCA distant metastases.^[^ ^5^ ^]^ Current rodent models of iCCA metastasis, including primary tumor cell xenograft and spontaneous tumor models, rarely metastasize to the lymph nodes or lung. On the other hand, the tumor cells tail‐vein injection model induces lung metastases. However, this model does not faithfully resemble the steps leading to cell dissemination from primary tumor cells. Therefore, developing appropriate in vivo metastasis models represents an urgent need for the current iCCA research.

Epithelial‐to‐mesenchymal transition (EMT) is a reversible dynamic process where epithelial cells gradually adopt mesenchymal cell properties.^[^ ^6^ ^]^ EMT is deeply involved in pathological processes, including embryogenesis, fibrosis development, and cancer progression.^[^ ^7^ ^]^ More specifically, the involvement of EMT‐like processes in iCCA has been proposed previously, mediating tumor cell migration and invasion from the primary tumor.^[^ ^8^ ^]^ Cells undergoing EMT progressively lose the expression of epithelial makers, such as E‐cadherin or ZO‐1 (TJP1), while gaining the expression of mesenchymal‐associated genes, such as Vimentin and N‐cadherin.^[^ ^9^ ^]^ EMT‐inducing transcription factors (EMT‐TFs), such as *Zeb, Twist, and Snail families*, can induce EMT directly, resulting in tumor development or metastasis in cancers. Recent studies have demonstrated that ZEB1 promotes iCCA progression by acquiring an EMT/cancer stem cell phenotype.^[^ ^10^ ^]^ However, the precise role of EMT on iCCA metastasis in vivo remains undefined.

Here, we describe a mouse *sgP19/kRAS*‐driven mixed iCCA model that exhibits metastasis to multiple organs, including the hilar lymph nodes, lung, and kidney. We further detected that the TGFβ/ZEB1 axis promotes the induction of EMT both in vivo and in vitro. Finally, we found that targeting EMT neither resulted in tumor inhibition nor led to metastasis restriction in this iCCA metastatic model. The data dispute the claim that EMT is an early and indispensable molecular event leading to tumor metastasis.

## 2 Results

### 2.1 sgP19/kRAS‐Derived Mixed iCCA Mouse Model Develops Metastasis

We generated the *sgP19/kRAS‐*induced iCCA model as described by Marco Seehawer *et al.*,^[^ ^11^ ^]^ in *C57/BL6* and *FVB/N* background mice. Elevated kRAS protein expression was validated in *sgP19/kRAS* tumor lesions in all mice (Figure S1A,B, Supporting Information). The deletion of the *P19* allele was confirmed using the TIDE assay (Figure S1C, Supporting Information). These data support that the tumors were driven by the injected plasmids. We found that tumor formation occurred slightly faster in *FVB/N* mice, with cystic lesions detectable by the third week post‐injection (Figure S2A,B, Supporting Information). By five to six weeks, all *FVB/N* mice developed high liver tumor burden, requiring euthanasia. In contrast, *C57BL/6* mice exhibited slower progression, with terminal tumor stages reached at 6 to 8 weeks (Figure S2A,B, Supporting Information).

**Figure 1:**
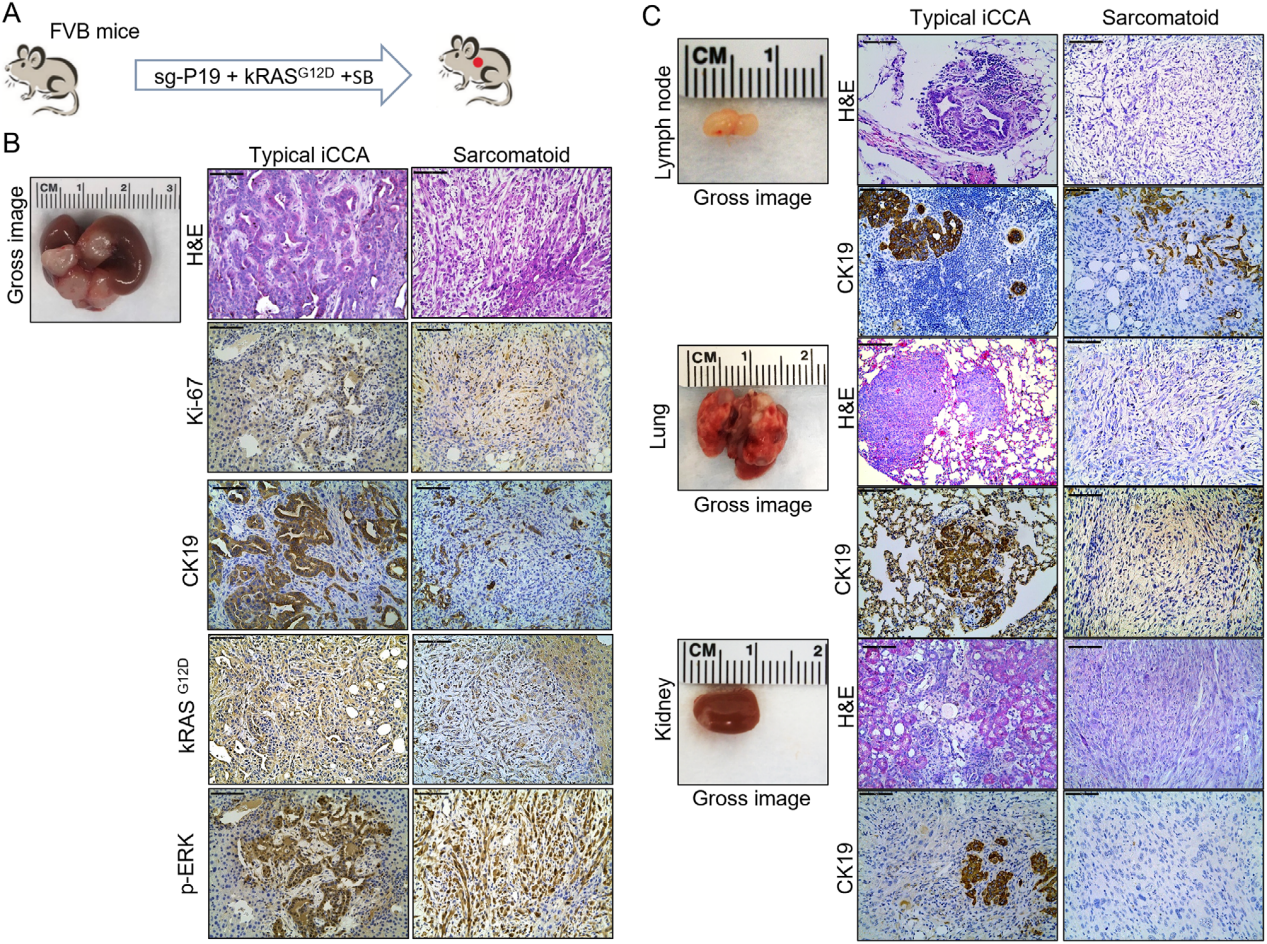
*sgP19/kRAS*‐derived iCCA mouse model develops tumor lesions in lymph nodes, lungs, and kidneys. A) Study design. FVB mice were injected with *sgP19/kRAS/SB* plasmids through HTVi. B) Gross image and H&E, CK19, Ki‐67, kRAS^G12D^, and p‐ERK staining in *sgP19/kRAS* mouse liver. C) Gross image and H&E, CK19 staining in lymph nodes, lungs, and kidneys of *sgP19/kRAS* mice. Abbreviations: sg‐P19, CRISPR/Cas9‐based gene deletion of p19; kRAS^G12D^, pCaggs‐kRAS^G12D^; SB, Sleeping beauty; HTVi, hydrodynamic tail vein injection; H&E, hematoxylin and eosin staining. Scale bar: 100 µm.

Upon careful histological examination, we noted typical iCCA lesions and sarcomatoid changes in the mouse liver (Figure 1A). The latter was characterized by pleomorphic spindle sarcomatoid cells (Figure 1B). We therefore refer to this mouse model as a mixed iCCA model. At the cellular levels, strong immunostaining for Ki‐67, kRAS^G12D^, and key downstream pathway protein p‐ERK was observed in both typical iCCA and sarcomatoid tumor cells (Figure 1B).

Studies have demonstrated that the poor prognosis of intrahepatic sarcomatoid cholangiocarcinoma is due to the frequent metastasis of sarcomatoid cells.^[^ ^12^ ^]^ Thus, we investigated the possible metastatic lesions in this mixed mouse iCCA model (Figure 1C). We collected various tissues from *sgP19/kRAS* injected mice in *C57/BL6* and *FVB/N* respectively, with 10 mice in each group. The incidence rate of liver tumors was 100%. Noticeably, all mice (≈100%) had tumors in the hilar lymph nodes (Figure 1C and Table S1, Supporting Information). Tumors had invaded the lung in 5 of the 10 mice. Moreover, we revealed tumors in the kidney in ≈30% of *sgP19/kRAS* mice. Equivalent results were found in *C57/BL6* and *FVB/N* mice. Similar to the primary liver tumors, both typical iCCA and sarcomatoid lesions were detected in the hilar lymph nodes, lungs, and kidneys (Figure 1C and Table S1, Supporting Information).

Next, we performed lineage tracing experiments to exclude the possibility that the “distant metastasis” detected in the hilar lymph nodes and other distal sites was induced by occasional plasmids' leakage to these organs. In brief, stochastic multicolor

**Figure 2:**
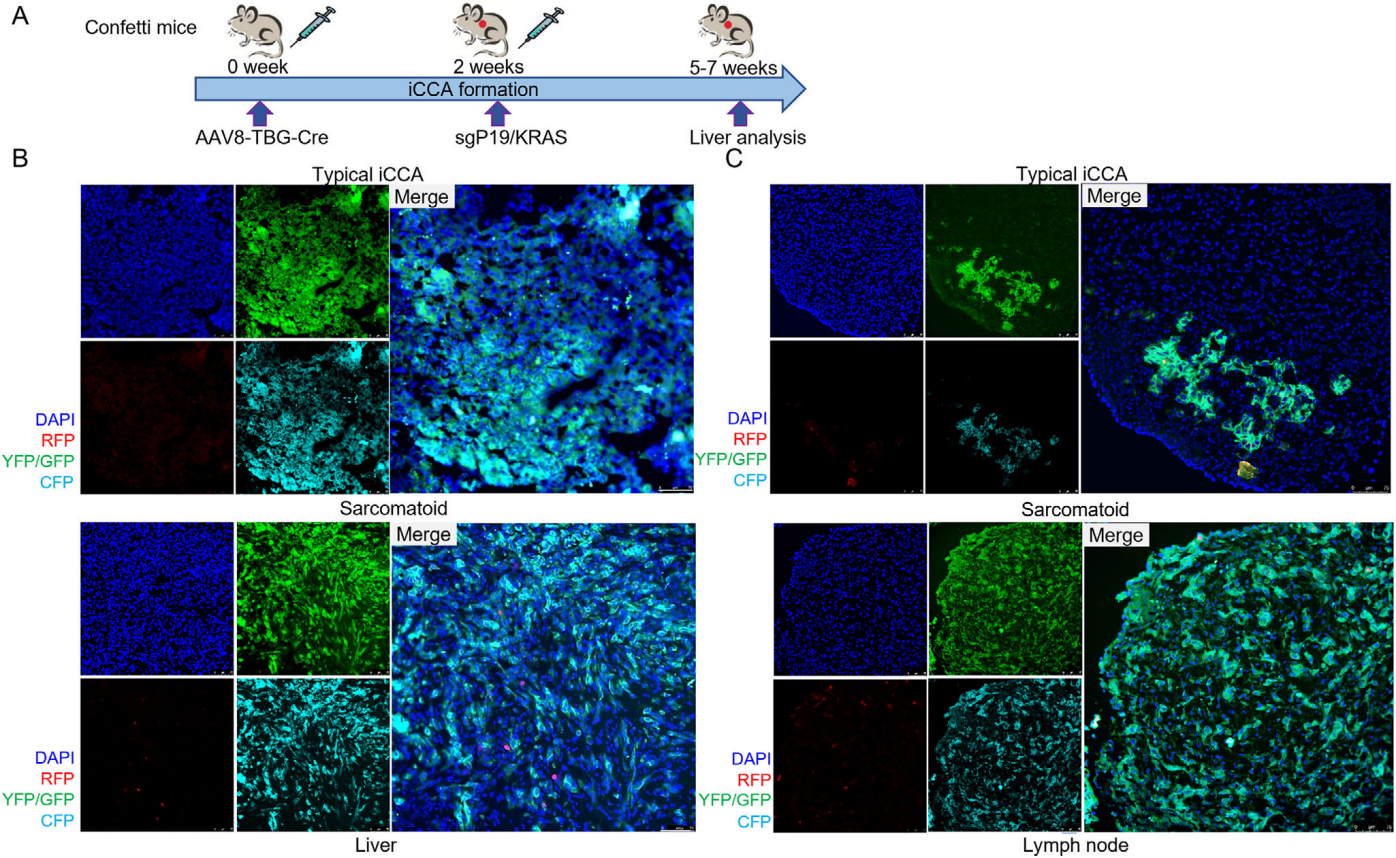
*sgP19/kRAS*‐derived iCCA mouse models induce metastasis. A) Study design. Four‐week‐old Confetti mice were injected with AAV8‐TBG‐Cre through the HTVi. The plasmid mixture of *sgP19/kRAS* was injected by hydrodynamic tail vein injection 2 weeks after lentivirus injection. Mice were sacrificed when moribund. Tumors were analyzed by fluorescence microscope. The fluorescently labeled hepatocytes could be detected in both typical iCCA and sarcomatoid iCCA in liver B) and lymph node C) tumors. Scale bars: 75 µm.

Cre‐reporter R26R‐confetti homozygous mice (that will be referred to as “Confetti mice”) were used (Figure 2A). Hepatocyte‐specific AAV8‐TBG‐Cre was injected into Confetti mice 2 weeks before hydrodynamic injection. Once the Cre‐recombinase is activated, the expression of the multicolor fluorescent proteins is triggered in mouse hepatocytes as previously described.^[^ ^13^ ^]^ If the tumors were developed in the labeled hepatocytes, the expression of fluorescent proteins would be maintained in tumor cells, although different cell lineages may be generated in these tumor cells. As expected, liver tumors found in *sgP19/kRAS* injected mice originated from hepatocytes, as they exhibited the fluorescent protein expression in typical iCCA and sarcomatoid tumor lesions (Figure 2B). Notably, the fluorescent proteins were also detected in lymph nodes, either in typical iCCA or sarcomatoid tumor lesions (Figure 2C), indicating the tumors from remote organs originated from hepatocytes. Therefore, distant metastasis was confirmed in the *sgP19/kRAS* mixed murine iCCA model.

The data indicate that the *sgP19/kRAS*‐derived mixed iCCA mouse model develops metastasis in distant organs, such as hilar lymph nodes, lungs, and kidneys.

### 2.2 EMT is Induced in the sgP19/kRAS Mixed iCCA Model

**Figure 3:**
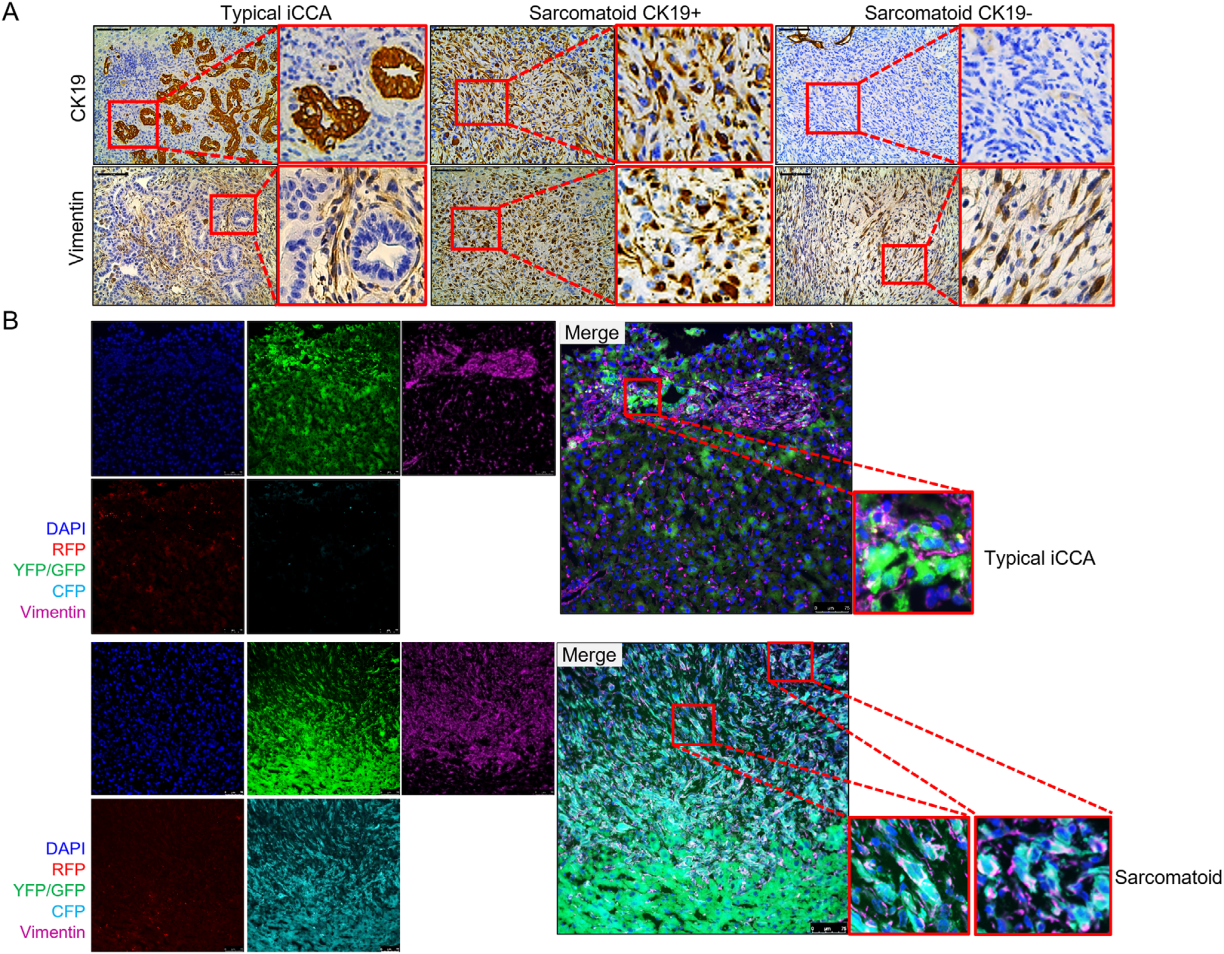
EMT occurs in liver tumor lesions of *sgP19/kRAS* mice. A) CK19 and Vimentin staining in *sgP19/kRAS* tumor lesions. B) Vimentin‐positive cells in sarcomatoid *sgP19/kRAS* tumors overlap with fluorescent proteins in Confetti reporter mice, while Vimentin staining did not overlap with the fluorescently labeled hepatocytes in typical iCCA tumor lesions. Scale bar: 100 µm (A); 75 µm (B).

Since EMT is an early metastasis event required for tumor cell migration and invasion from the primary tumor,^[^ ^6^ ^]^ we examined whether this phenotype occurs in the *sgP19/kRAS* mixed iCCA model. We performed a detailed analysis of epithelial and mesenchymal markers in liver tumor lesions. We found that iCCA tumor cells express epithelial markers, including CK19, CK7 and E‐Cadherin, whereas sarcomatoid tumor cells were negative for these markers, but instead showed positive staining of mesenchymal markers, including Vimentin, Smooth muscle actin (SMA), and S100A4 (Figures 3A and S3, Supporting Information).

We chose to use the epithelial marker, CK19, and the mesenchymal marker Vimentin for our subsequent analysis. Three distinct patterns emerged. In the first pattern, a subset of tumor lesions was positive for CK19 on tumor cells, while Vimentin immunoreactivity was limited to the surrounding cancer‐associated fibroblasts (CAFs) (Figure 3A left), thus representing the typical iCCA lesions. To confirm that Vimentin (+) cells were CAFs, we re‐analyzed the slides from the lineage tracing experiments using immunofluorescent staining. We found that tumor cells were positive for the fluorescent signals, and they did not overlap with those signals from Vimentin (+) cells, supporting that the Vimentin (+) cells were indeed CAFs but not tumor cells (Figure 3B). To substantiate this result, we performed Immunohistochemistry (IHC) staining of CK19, Ki67, and Vimentin on the consecutive sections of the mouse liver tumors (Figure S4, Supporting Information) as we reasoned that CAFs are derived from hepatic stellate cells with low proliferative activity. Indeed, we could see that in the typical iCCA lesions, Ki67 (+) cells were limited to CK19 (+) iCCA cells, whereas Vimentin (+) cells were Ki67 (‐) (Figure S4, Supporting Information). The data are consistent that these Vimentin (+) cells are non‐proliferating CAFs.

A second pattern consisted of Vimentin (+) but CK19(‐) sarcomatoid tumors (Figure 3A right). Vimentin immunofluorescence on the lineage tracing slides revealed that the fluorescent signaling overlapped with Vimentin staining (Figure 3B). The data suggested that these Vimentin (+) cells derived from hepatocytes, and they gained mesenchymal character and became Vimentin (+) sarcomatoid tumor cells during tumor development. Using IHC staining of CK19, Ki67, and Vimentin on the consecutive sections of the mouse liver tumors, we found that these Vimentin (+) cells in the sarcomatoid lesions were Ki67 (+), supporting that they were indeed proliferating tumor cells (Figure S4, Supporting Information).

Intriguingly, for the third pattern, some sarcomatoid tumor cells stain positive for both Vimentin and CK19 (Vimentin (+); CK19 (+)) (Figure 3A middle and Figure S5, Supporting Information). These results were consistent with EMT progression, i.e, these cells started to gain mesenchymal markers before losing their epithelial markers. The presence of these cells provides strong evidence for EMT.

Finally, we analyzed the lesions in lymph node metastasis from these mice. Both typical iCCA and sarcomatoid tumors could be noted (Figure S6, Supporting Information), indicating the occurrence of EMT in metastasis lesions.

Overall, our data demonstrate that EMT was induced in a subset of liver tumors in the *sgP19/kRAS* model.

### 2.3 TGFβ Pathway Regulates EMT in *sgP19/kRAS* Mice

**Figure 4:**
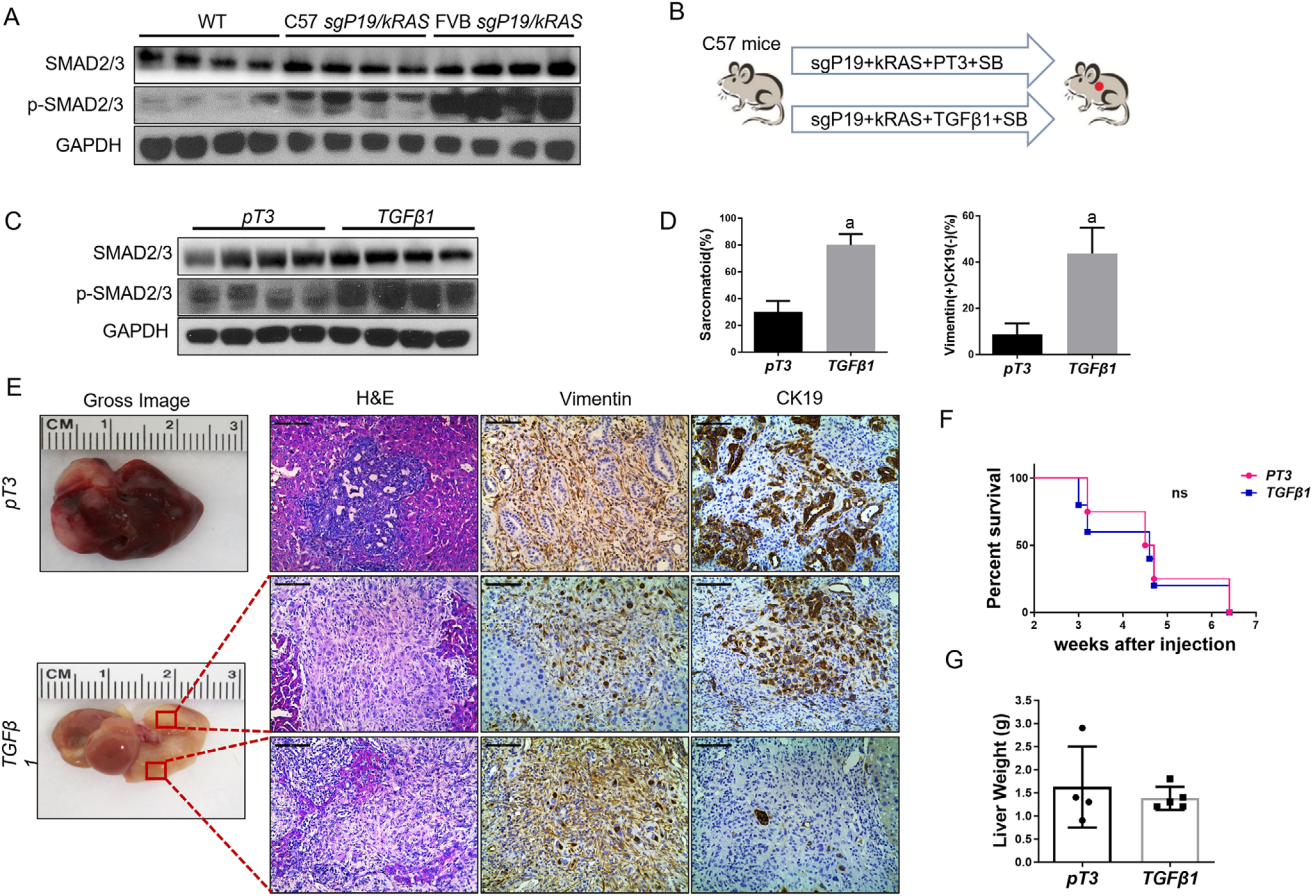
TGFβ1 promotes EMT in the *sgP19/kRAS*‐induced iCCA model. A) Western blot results showing p‐SMAD2/3 expression of wild‐type (WT) normal liver, *sgP19/kRAS* FVB, and C57 tumor tissues. GAPDH was used as a loading control. B) Study design. C57 mice were injected with *sgP19/kRAS/PT3* or *sgP19/kRAS/TGFβ1* plasmids, respectively. Mice were monitored and sacrificed when moribund. C) Western blot results showing SMAD2/3 and p‐SMAD2/3 expression in *sgP19/kRAS/PT3* and *sgP19/kRAS/TGFβ1* tumor tissues. D) Percentage of sarcomatoid and Vimentin + CK19‐ staining tumor lesions. E) Representative H&E and immunohistochemical staining images of Vimentin and CK19 in *sgP19/kRAS/PT3* (up) and *sgP19/kRAS/TGFβ1* tumor lesions. Two lesions were presented in the *sgP19/kRAS/TGFβ1* tumor lesion, Vimentin+ CK19+ (middle) and Vimentin+ CK19‐ (low), respectively. F) Survival curve and G) liver weight of C57 mice injected with *sgP19/kRAS/PT3* (n = 4) or *sgP19/kRAS/TGFβ1* (n = 5). Student's t‐test: at least P < 0.05; a, vs. pT3; *sgP19/kRAS/PT3* mice. Scale bar: 100 µm.

Many studies have shown that the TGFβ signaling pathway promotes the EMT process, thus inducing the invasion and metastasis of malignant tumors.^[^ ^14^, ^15^ ^]^ We next investigated whether the promotion of EMT in the *sgP19/kRAS* model is triggered by the TGFβ signaling pathway. Using the proportion of sarcomatoid tumor lesions and Vimentin (+) CK19 (‐) tumor cells as markers for EMT progression, we found that the tumors in *FVB/N* mice contained a significantly higher proportion of Vimentin (+) CK19 (‐) tumor cells. which demonstrated that *sgP19/kRAS*‐induced EMT is much more prominent in *FVB/N* mice than in *C57BL/6* mice (Figure S7, Supporting Information). Consistently, the activation of the TGFβ cascade was more prominent in *FVB/N sgP19/kRAS* liver tumors than in *C57BL/6 sgP19/kRAS*, with the increased expression of p‐Smad2/3 (Figure 4A). To test the role of the TGFβ pathway in vivo, we examined whether TGFβ overexpression suffices to promote EMT induction. Thus, the *TGFβ1* cDNA was cloned into the EF1α vector and was co‐injected with *kRAS*, *sgP19*, and Sleeping beauty transposase (SB) into the *C57BL/6* mouse liver (Figure 4B). Activation of the TGFβ1 cascade promoted the level of EMT progression as indicated by increasing the protein levels of phosphorylated Smad2/3 and the region of sarcomatoid tumor lesions and the proportion of Vimentin (+) CK19 (‐) tumors in *sgP19/kRAS C57BL/6* mice (Figure 4C,D). Despite the increased EMT, overexpression of TGFβ1 did not significantly influence the tumor development, and all *sgP19/kRAS* mice injected with pT3‐EF1α empty vector or TGFβ1 developed high tumor burden by ≈7 weeks after injection (Figure 4E,F). In addition, the two mouse cohorts demonstrated similar liver tumor burden as measured by total liver weight (Figure 4G).

These data suggested that the TGFβ pathway enhances the EMT ability of iCCA cells in vivo while not influencing tumor development in *sgP19/kRAS* mice.

### 2.4 TGFβ Induces EMT by Activating the ZEB1 Pathway

**Figure 5:**
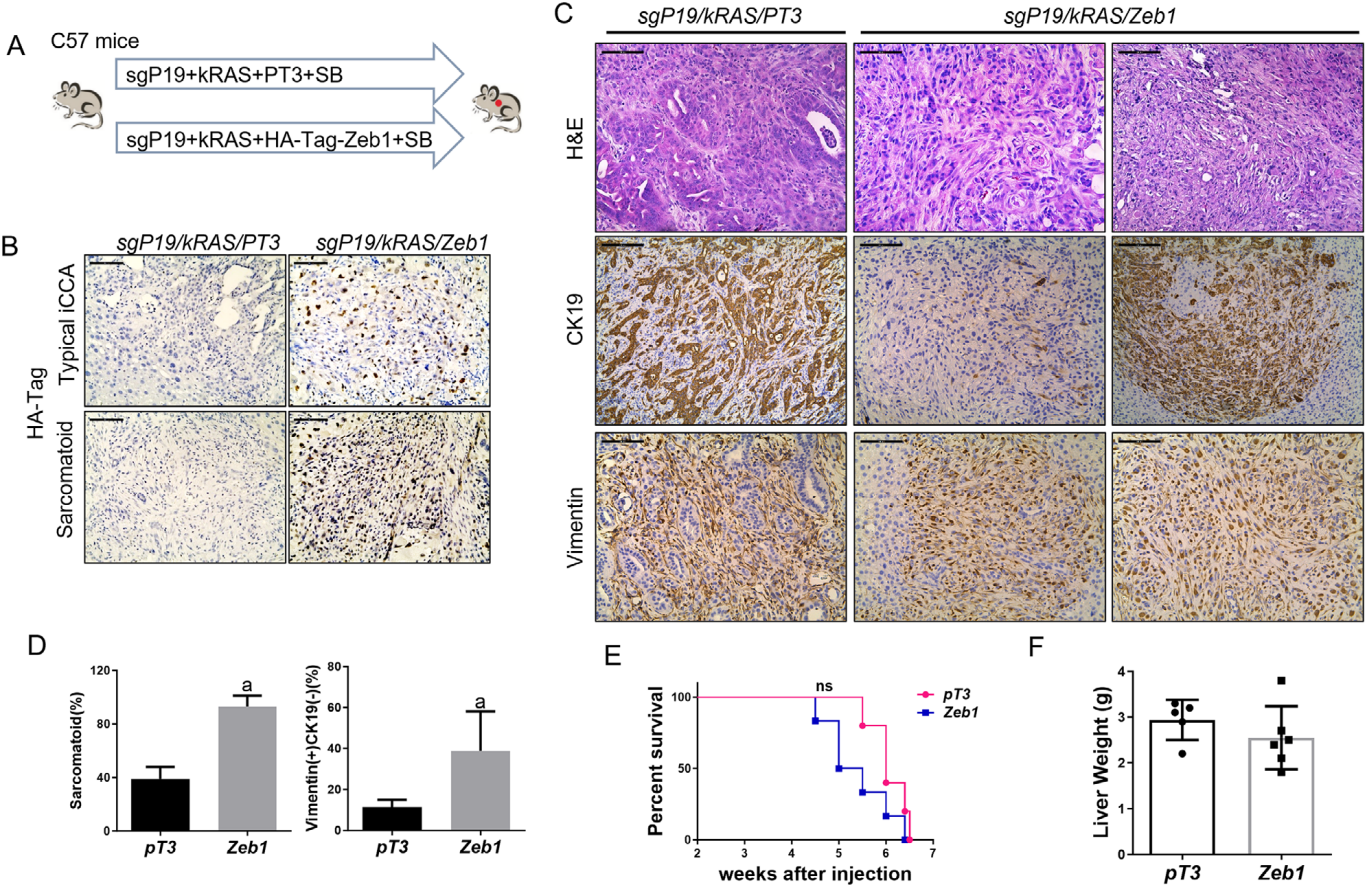
ZEB1 promotes EMT in the *sgP19/kRAS*‐induced iCCA model. A) Study design. C57 mice were injected with *sgP19/kRAS/PT3* or *sgP19/kRAS/HA‐tag‐Zeb1* plasmids, respectively. Mice were monitored and sacrificed when moribund. B) HA‐tag staining in *sgP19/kRAS/PT3* and *sgP19/kRAS/Zeb1* tumor tissues. C) Gross image and H&E, Vimentin, CK19 staining in *sgP19/kRAS/PT3* and *sgP19/kRAS/Zeb1* tumor lesions. D) Percentage of sarcomatoid and Vimentin+ CK19‐ staining tumor lesions. E) Survival curve and F) liver weight of C57 mice injected with *sgP19/kRAS/PT3* (n = 5) or *sgP19/kRAS/Zeb1* (n = 6). Student's t‐test: at least P < 0.05; a, vs. pT3; *sgP19/kRAS/PT3* mice. Scale bar: 100 µm.

Numerous studies have shown that molecular reprogramming during EMT is triggered and orchestrated directly by modulating the main EMT‐activating transcriptional factors, including the zinc finger E‐box binding homeobox proteins (ZEB), Snail family, and Twist family.^[^ ^16^ ^]^ Thus, we investigated whether TGFβ mediated tumor cell EMT depends on these transcription factors. RT‐qPCR was used to determine the mRNA expression of *Snail1*, *Snail2* (*Slug*), *Twist1*, and *Zeb1* in tumors of *C57BL/6* and *FVB/N sgP19/kRAS* mice (Figure S8, Supporting Information). All these EMT‐TFs were upregulated in *C57BL/6* and *FVB/N* mice tumor lesions compared to the normal liver. Moreover, as expected, the mRNA levels of *Snail1*, *Twist1*, and *Zeb1* were much higher in *FVB/N sgP19/kRAS* mice than in *C57BL/6* mice (Figure S8, Supporting Information), consistent with the significantly more Vimentin (+) CK19 (‐) EMT lesions in *sgP19/kRAS FVB/N* mice. Therefore, in subsequent studies, three transcription factors, namely *Snail1*, *Twist1*, and *Zeb1*, were selected for research. To further elucidate whether EMT triggered in the *sgP19/kRAS* model depends mainly on the regulation of *Snail1*, *Twist1*, or *Zeb1*, we overexpressed each of these genes separately in *sgP19/kRAS C57BL/6* mice, which has a lower percentage of Vimentin (+) CK19 (‐) EMT lesions. In brief, *C57BL/6* mice were hydrodynamically injected with *kRAS*, *sgP19*, and SB plasmids, combined with *Snail1* (*sgP19/kRAS/Snail1*), *Twist1* (*sgP19/kRAS/Twist1*), or *Zeb1* (*sgP19/kRAS/Zeb1*) plasmids (Figure  5A; Figure S9A, Supporting Information). Additional mice were injected with *kRAS*, *sgP19*, *SB*, and the *pT3‐EF1α* empty vector (*sgP19/kRAS/PT3*). Neither *Snail1* nor *Twist1* overexpression promoted the sarcomatoid tumor development or increased the area of Vimentin (+) CK19 (‐) tumor lesions (Figure S9B,C, Supporting Information). In contrast, overexpressing *Zeb1* (HA‐tagged) significantly increased the area of both sarcomatoid tumor lesions and Vimentin (+) CK19 (‐) tumors (Figure 5B–D), promoting the development of EMT in *sgP19/kRAS* tumor tissues. In addition, consistent with the TGFβ1 in vivo data, the induction of EMT by *Zeb1* overexpression neither accelerated the tumor development nor increased the tumor burden in *sgP19/kRAS* mice (Figure 5E,F). To confirm that ZEB1 promotes EMT in human iCCA cells, we transfected the KMCH iCCA cell line with *Zeb1*. Immunoblotting demonstrated augmented expression of ZEB1 after *Zeb1* transfection in KMCH cells (Figure S10A, Supporting Information). The epithelial marker E‐cadherin and CK19 expression were substantially lower upon *ZEB1* overexpression (Figure S10A, Supporting Information). Furthermore, overexpression of *TGFβ1* upregulated the expression of ZEB1 and promoted EMT induction, indicated by the decreased expression of E‐cadherin and CK19 (Figure S10B, Supporting Information). Finally, we examined *ZEB1* mRNA expression using the NCI iCCA gene expression dataset.^[^ ^17^, ^18^ ^]^ We found that *ZEB1* expression was strongly positively correlated with the expression of mesenchymal markers, such as *VIM*, *TWIST1*, and *ZEB2*, and negatively correlated with the epithelial marker *CDH1* (Figure S11A, Supporting Information). *ZEB1* expression also correlated with multiple TGFβ pathway target genes, such as *SMAD4, TGFb1I1, TGFBR2, TGFBR3*, *and TGFB3* (Figure S11B, Supporting Information), supporting the correlation between *ZEB1* and activated *TGFβ* in iCCA.

These results show that TGFβ may promote EMT in iCCA by inducing ZEB1 expression.

### 2.5 The Effect of EMT Inhibition in the Progression and Metastasis of *sgP19/kRAS* iCCA In Vivo

Next, we investigated the role of EMT in the progression and metastasis in the *sgP19/kRAS* model. Thus, we thoroughly analyzed the iCCA and EMT lesions in the liver and LN or lung metastases in *sgP19/kRAS FVB/N* mice (Figure S12, Supporting Information). If EMT is a driving factor for tumor metastasis, one would predict that there are more EMT lesions in the metastatic tumors. In contrast to this hypothesis, we found that the percentage of the area of both sarcomatoid tumor lesions and Vimentin (+) CK19 (‐) tumors was much lower in LN/Lung metastasis tumors than that in *sgP19/kRAS* primary tumors (Figure S12, Supporting Information), suggesting EMT is not a critical driver in the *sgP19/kRAS* model.

**Figure 6:**
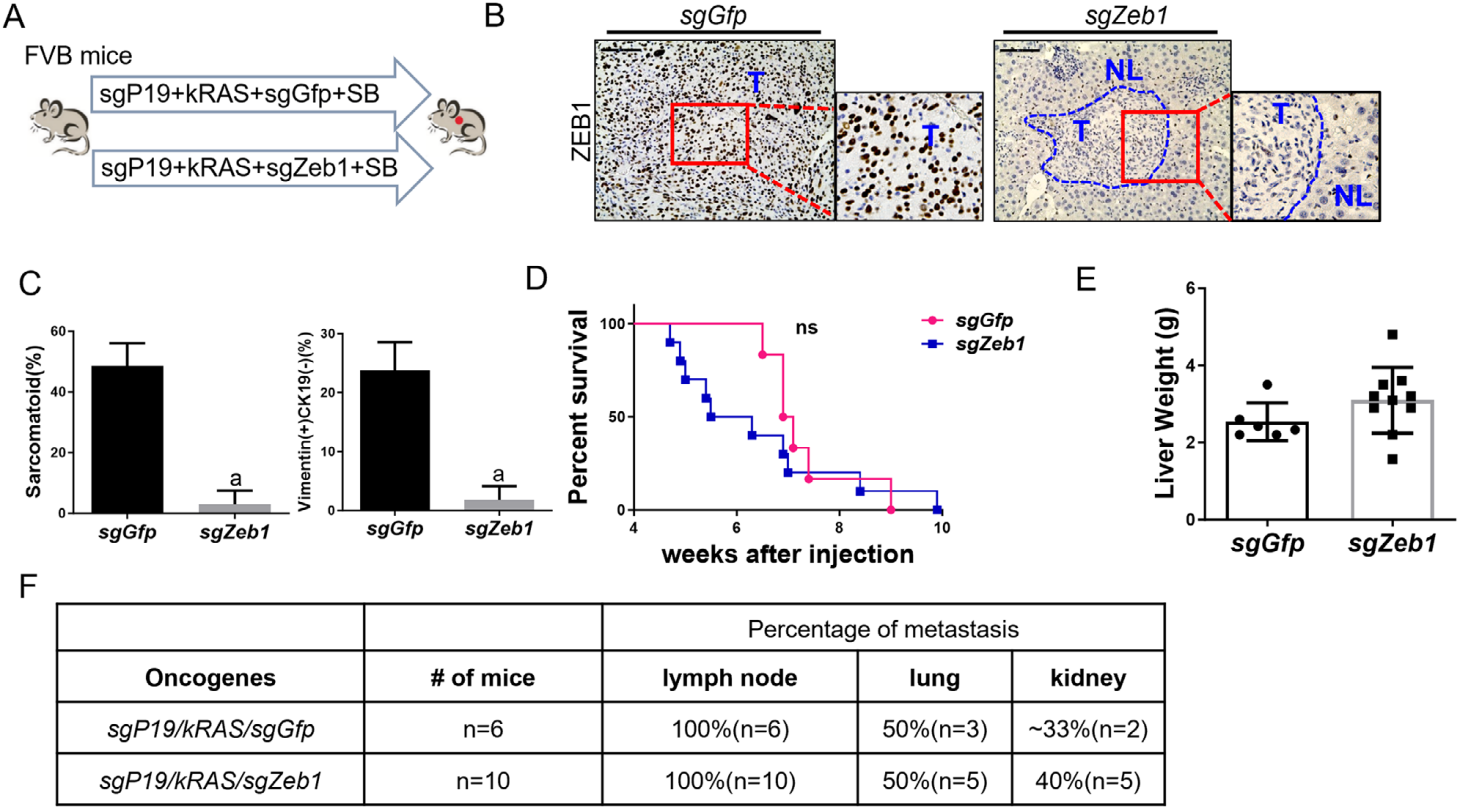
Deletion of ZEB1 inhibits EMT without delaying carcinogenesis or suppressing metastasis in the *sgP19/kRAS*‐induced iCCA mice model. A) Study design. FVB mice were injected with *sgP19/kRAS/sgGfp* or *sgP19/kRAS/sgZeb1* plasmids, respectively. Mice were monitored and sacrificed when moribund. B) ZEB1 staining in *sgP19/kRAS/sgGfp* and *sgP19/kRAS/sgZeb1* tumor tissues. C) Percentage of sarcomatoid and Vimentin+ CK19‐ staining tumor lesions. D) Survival curve showing that *Zeb1* knockout does not prolong *sgP19/kRAS* mouse survival. E) Liver weight of FVB mice injected with *sgP19/kRAS/sgGfp* (n = 6) or *sgP19/kRAS/sgZeb1* (n = 10). F) Summary of the percentage of metastasis in lumph node, lung, and kidney of *sgP19/kRAS/sgGfp* or *sgP19/kRAS/sgZeb1* mice. Student's t test: at least *P < 0.05*; a, vs. *sgP19/kRAS/sgGfp* mice. Scale bar: 100 µm.

To verify our conclusion, we examined whether EMT inhibition by suppressing the TGFβ pathway or *Zeb1* knockout could prevent metastasis in *sgP19/kRAS* mice. Therefore, we applied CRISPR/Cas9 based gene deletion of Zeb1 (sgZeb1) in vivo. We designed three independent guide RNA sequences against mouse *Zeb1*, which were then subcloned into the pX330 vector (*sgZeb1*). Subsequently, we co‐injected *sgZeb1* with *sgP19/kRAS* and *SB* plasmids into the mice (Figure 6A). All three *sgZeb1* constructs were able to inhibit ZEB1 protein expression in the *sgP19/kRAS* mixed model (*sgZeb1‐1*, Figure 6B and *sgZeb1‐2,1‐3*, Figure S13A, Supporting Information). Thus, we randomly chose one of these three plasmids (*sgZeb1‐1*) to demonstrate the function of EMT in the following experiments. In brief, we injected *sgZeb1* together with *kRAS* and *sgp19* (*sgP19/kRAS/sgZeb1*) in the *FVB/N* background, which has a higher EMT development compared with that of *C57BL/6* mice. Additional mice were injected *kRAS*, *sgP19* with *sgGfp* (in pX330 plasmid) as a control (*sgP19/kRAS/sgGfp*) (Figure 6A). As expected, loss of ZEB1 expression could be easily visualized in *sgP19/kRAS/sgZeb1* tumor lesions (Figure 6B). Histopathological analysis revealed that the area of both sarcomatoid tumors and Vimentin (+) CK19 (‐) tumor lesions were substantially decreased in *sgP19/kRAS/sgZeb1* mice (Figure 6C, Figure S13B, Supporting Information), indicating the inhibition of EMT upon *Zeb1* knockout. Both *sgP19/kRAS/sgGfp* and *sgP19/kRAS/sgZeb1* injected mice developed a lethal burden of liver tumors 10 weeks after injection (Figure 6D). The two cohorts of mice demonstrated similar liver tumor burden as measured by total liver weight (Figure 6E). The metastasis rate of different organs was also summarized (Figure 6F), showing that there was no substantial difference between *sgP19/kRAS/sgZeb1* and *sgP19/kRAS/sgGfp* mice in the percentage of metastasis in lymph node (≈100%), lungs (≈50%), and kidneys (≈30‐40%), indicating that EMT inhibition by *Zeb1* ablation had no influence in metastasis in the *sgP19/kRAS* model (Figure 6F).

**Figure 7:**
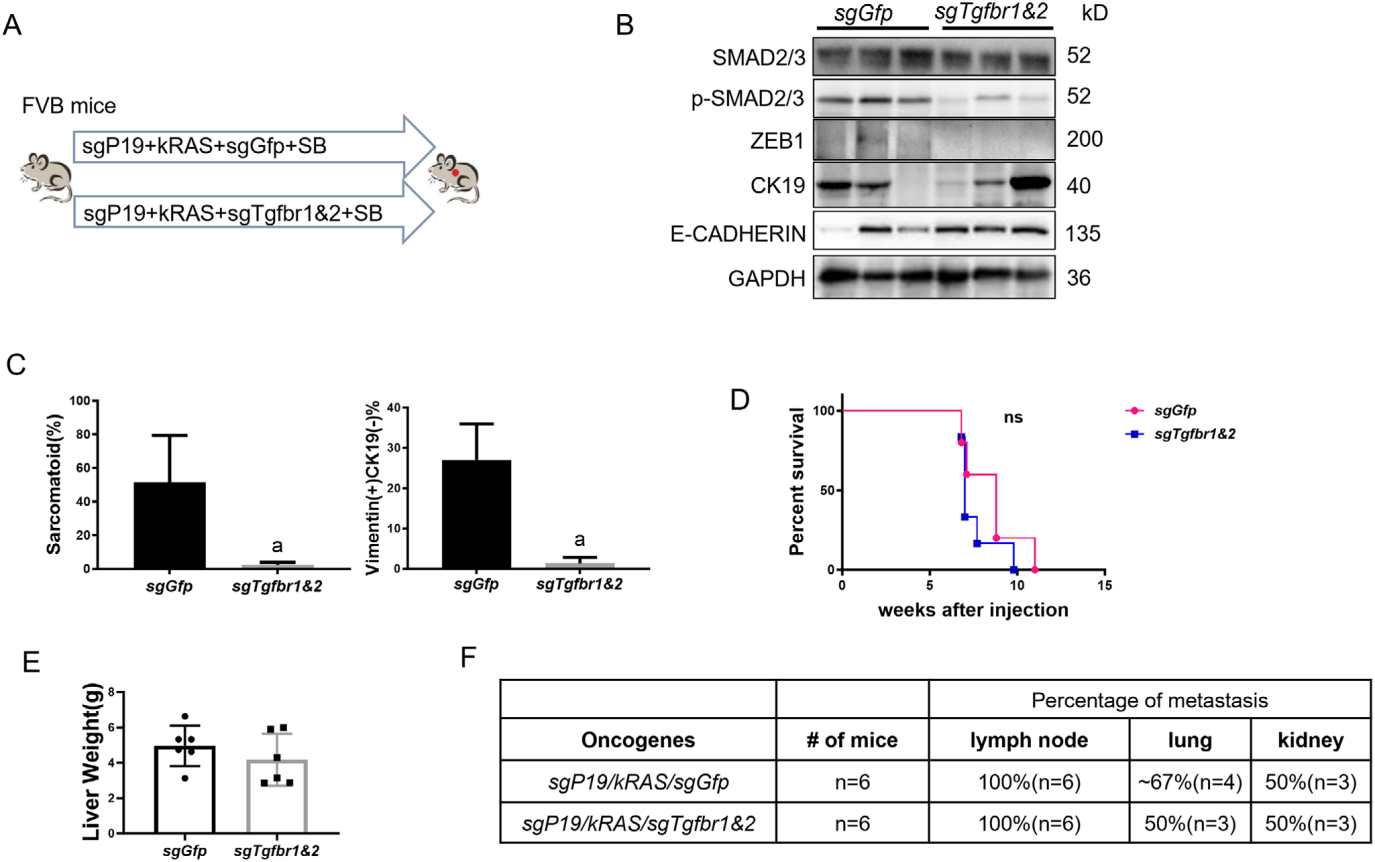
Deletion of Tgfbr1&2 inhibits EMT without delaying carcinogenesis or suppressing metastasis in *sgP19/kRAS*‐induced iCCA mice model. A) Study design. FVB mice were injected with *sgP19/kRAS/sgGfp* or *sgP19/kRAS/sgTgfbr1&2* plasmids, respectively. Mice were monitored and sacrificed when moribund. B) Western blot results showing knockout effect of *Tgfbr1&2, sgP19/kRAS* FVB tissues. GAPDH was used as a loading control. C) Percentage of sarcomatoid and Vimentin+ CK19‐ staining tumor lesions. D) Survival curve showing that TGFβ1 knockout does not prolong *sgP19/kRAS* mouse survival. E) Liver weight of FVB mice injected with *sgP19/kRAS/sgGfp* (n = 6) or *sgP19/kRAS/sgTgfbr1&2* (n = 6). F) Summary of the percentage of metastasis in lymph nodes, lungs, and kidneys of *sgP19/kRAS/sgGfp* and *sgP19/kRAS/sgTgfbr1&2* mice. Student's t‐test: at least *P < 0.05*; a, vs. *sgP19/kRAS/sgGfp* mice.

TGFβ functions via receptor 1 (TGFβR1) and 2 (TGFβR2). sgRNAs against mouse *Tgfbr1* and *Tgfbr2* were subcloned into the pX330 vector simultaneously for in vivo gene deletion (*sgTgfbr1&2*) (Figure S14A, Supporting Information). Next, *sgTgfbr1&2* were injected together with *kRAS* and *sgP19* (*sgP19/kRAS*/*sgTgfbr1&2*) (Figure 7A) in *FVB/N* background mice. The expression levels of *Tgfbr1* and *Tgfbr2* were detected by Western blotting and immunohistochemistry in the *sgP19/kRAS*/*sgTgfbr1&2* and *sgP19/kRAS*/*sgGFP* models (Figure S15, Supporting Information), supporting that *sgTgfbr1&2* effectively inhibited TGFβR1 and TGFβR2 expression in the tumor cells. Furthermore, *sgTgfbr1&2* could effectively inhibit the activation of the TGFβ pathway in the *sgP19/kRAS* model, as verified by the downregulation of p‐SMAD2/3 levels (Figure 7B). Similar to the phenotype of the *Zeb1* knockout *sgP19/kRAS* model, inhibition of the TGFβ pathway substantially decreased the area of both sarcomatoid tumors and Vimentin (+) CK19 (‐) tumor lesions in *sgP19/kRAS*/*sgTgfbr1&2* mice (Figure 7C, Figure S14B, Supporting Information), indicating the inhibition of EMT in these mice. However, mice survival and tumor burden were not statistically significant different between *sgP19/kRAS*/*sgTgfbr1&2* and *sgP19/kRAS/sgGfp* mice (Figure 7D‐E). Moreover, the EMT suppression induced by TGFβ inhibition had no effects on metastasis as same as *Zeb1* knockout in the *sgP19/kRAS* model, indicated by the similar percentage of metastasis in lymph nodes (≈100%), lungs (≈50‐70%), and kidneys (≈50%) in *sgP19/kRAS*/*sgTgfbr1&2* and *sgP19/kRAS/sgGfp* mice (Figure 7F).

SMAD7 belongs to the subclass of inhibitory SMADs that are antagonists of the TGFβ signaling.^[^ ^19^ ^]^ Thus, we overexpressed HA‐tagged *Smad7* in *sgP19/kRAS* mice with a *FVB/N* background mice to inhibit the TGFβ pathway. We found that overexpression of *Smad7* successfully inhibited EMT in *sgP19/kRAS* mouse liver tumors. However, it did not affect tumor formation or metastasis (Figure S16, Supporting Information).

In summary, these data revealed that the EMT induced by the TGFβ/ZEB1 axis does not regulate tumor development or distant metastasis in the *sgP19/kRAS* mixed iCCA model.

## 3 Discussion

EMT is a crucial element in tumor progression. The role of the EMT in tumor metastasis has been controversial in the academic circle in recent years.^[^ ^20^ ^]^ Most of the initial studies believed that EMT promoted the invasion and metastasis of tumor cells.^[^ ^21^, ^22^ ^]^ Still, most of the studies were only conducted at the level of in vitro experiments. In recent years, several reports described the occurrence of metastases formed by cells that did not undergo EMT. Currently, EMT is considered a critical inducer of tumor metastasis in iCCA.^[^ ^23^, ^24^ ^]^ However, most related studies have been conducted in vitro and cell ectopic transplantation tumor models, lacking direct in vivo experimental evidence. Therefore, further investigation is required to determine whether the iCCA metastasis requires EMT of tumor cells and whether the occurrence of EMT is the inducer of tumor metastasis.

In the present study, we show a 100% incidence of spontaneous extrahepatic metastasis, affecting lymph nodes, lungs, and kidneys in an iCCA model induced by the overexpression of mutant *kRAS* gene and the knockout of cell cycle suppressor protein *P19* using hydrodynamic injection (Figure 1).^[^ ^25^ ^]^ At the same time, lineage tracing technology was applied to confirm that the metastatic tumor cells originated from liver tumors and were not due to the overexpression of plasmids in extrahepatic organs, such as lymph nodes, lungs, or kidneys (Figure 2). This finding is consistent with clinical findings. Indeed, previous investigations revealed that iCCA patients with *kRAS* mutations are more likely to develop lymph node metastases.^[^ ^26^ ^]^

Notably, in the *sgP19/kRAS* iCCA model, we detected the occurrence of EMT, manifested by the transformation of bile duct epithelial tumor cells into fusiform tumor cells (sarcomatoid tumor type). Through lineage tracing technology, we found that tumor cells derived from mature hepatocytes simultaneously expressed the mesenchymal marker Vimentin. EMT occurrence was also detected in metastatic tumors (Figure S6, Supporting Information). This is the first time EMT has been detected in an iCCA in vivo metastatic tumor model.

TGFβ pathway is central to inducing EMT in several tissue types.^[^ ^27^, ^28^, ^29^ ^]^ Thus, we activated the TGFβ signaling pathway by delivering the TGFβ1 plasmid into *sgP19/kRAS* mice. As expected, activation of TGFβ1 did promote EMT in vivo. However, overexpression of TGFβ1 did not significantly augment tumor development. In addition, the EMT‐TFs have a significant role in orchestrating EMT programmers. Thus, we overexpressed *Snail1*, *Twist1*, and *Zeb1* separately in *sgP19/kRAS C57BL/6* mice. Notably, the development of EMT was promoted by overexpressing *Zeb1* in *sgP19/kRAS* tumor tissues, but *Zeb1* knockout did not significantly delay or accelerate the tumor process and metastasis. Similar negative results were obtained following the knockout of TGFβ1.

The present study builds on these previous works by offering several insights. First, we presented for the first time a *sgP19/kRAS*‐driven iCCA mouse model with 100% incidence of spontaneous extrahepatic metastasis. To some extent, this fills the gap that there is currently no reliable iCCA mouse metastasis model. Second, we first discovered'he the occurrence of EMT in mouse iCCA. This discovery provides a new horizon for the study of EMT in vivo. Third, EMT induced by the TGFβ/ZEB1 axis does not influence tumor development or distant metastasis in the *sgP19/kRAS* model. The data suggest that EMT is dispensable during tumor metastasis, at least in this murine iCCA model. Additional studies in vivo using novel models are required to better define EMT's functional role during tumor metastasis.

The animal model described in this study provides the basis for further research of iCCA metastasis. Furthermore, the occurrence of EMTs described here, although limited in that it was only validated in the *sgP19/kRAS* model, provides novel insights into the role of EMTs in tumor metastasis and a new idea for further study of EMT.

## 4 Experimental Section

### Constructs and Reagents

Constructs applied in this study include CRISPR/Cas9‐based gene deletion of p19(sgP19), pCaggs‐kRAS^G12D^ (kRAS, Human), Sleeping beauty (SB), pT3‐EF1α (PT3), pT3‐EF1a‐TGFβ1 (TGFβ1， mouse), pT3‐EF1a‐HA‐Tag‐ZEB1 (HA‐Tag‐Zeb1), pT3‐EF1α‐HA‐Smad7 (Smad7, Human), pT3‐EF1α Flag‐Snail1 (Snail1, human), pT3‐EF1α‐Twist (Twist, Mice), CRISPR/Cas9‐based gene deletion of GFP (sgGFP), CRISPR/Cas9‐based gene deletion of Zeb1 (sgZeb1) and CRISPR/Cas9‐based gene deletion of Transforming Growth Factor Beta Receptor 1 and 2 (sgTgfbr1&2). SB, kRAS^G12D^ transposon plasmids, and sgP19 were provided by L. Zender, University of Tübingen, Germany. ZEB1 (human; cat. #42100), pBABE‐puro‐mTwist (#1783) and pcmv5‐smad7‐HA (#11733) were obtained from Addgene. ZEB1 was also cloned into the pLenti‐puro vector for in vitro studies. Flag‐SNAI1 (human; cat. #16218) was obtained from Addgene. It was used as the template to generate pT3‐EF1α‐Flag‐SNAI1 (SNAI1), constructs via the Gateway cloning strategy. EGFP/plenti‐puro was obtained from Addgene (cat. #26431) and used as a control. Dosages of these plasmids and all model's animals numbers showed in the Table S5 (Supporting Information). All plasmids were purified utilizing the Endotoxin‐Free Maxiprep kit (Sigma–Aldrich, St. Louis, MO, USA).

### Cell Culture and In Vitro Studies

The KMCH iCCA cell line used for the in vitro studies was a generous gift by Dr. Gregory J. Gores (Mayo Clinic, Rochester, MN, USA). Cell lines were validated (Genetica DNA Laboratories, Burlington, NC, USA) and maintained as monolayer cultures in Dulbecco's modified Eagle medium with 10% fetal bovine serum (FBS; Gibco, Grand Island, NY, USA), 100 U/mL penicillin, and 100 g/mL streptomycin (Gibco). Transfections with lentivirus assay were performed as described before in detail.^[^ ^30^ ^]^

### Statistical Analysis

The Prism 7.0 software (GraphPad, San Diego, CA) was used to analyze the data presented as Means ± SD. Comparisons between the two groups were performed with a two‐tailed unpaired t‐test when the dataset achieved a Gaussian distribution or the non‐parametric test when the sample size was small. Welch correction and linear regression were applied when necessary. Kaplan–Meier method and log‐rank test were used for survival analysis. Multi‐variate Cox regression analysis using Statistical Package for Social Science Software (SPSS, version 16.0, Chicago, IL, USA) was applied to compare the hazard ratio groups with confounding variables taken into account. P values < 0.05 were considered statistically significant.

Additional detailed materials and methods are available in the Supporting document.

## Conflict of Interest

The authors declare no conflict of interest.

## Author Contributions

J.W. and Z.X. equally contributed to this work. Jingwen Wang, Zijing Xu, Lei Xu, Lishan Wang, Jiahao Geng, Xue Wang, Meng Xu, and Daphne Superville generated data, key reagents, and samples. Jingwen Wang, Zijing Xu analyzed and interpreted the data. Jingwen Wang, Xinhua Song, Xin Chen, Matthias Evert, Diego F. Calvisi and Melissa Reeves wrote and/or revised the manuscript. Xin Chen, Xinhua Song, Diego F. Calvisi and Matthias Evert were involved in study design and obtaining funding. All authors have read and approved the manuscript.

## Supporting information



Supporting Information

## Data Availability

The data that support the findings of this study are available from the corresponding author upon reasonable request.
