# Supplementary material for: Uncoupling Metastasis and Epithelial‐to‐Mesenchymal Transition in sgP19/kRAS‐Driven Spontaneous Metastatic Liver Tumor Model
Source: Adv Sci (Weinh). 2025 Dec 7;13(8):e14198. doi: 10.1002/advs.202514198 (PMC12884727; doi:10.1002/advs.202514198)
Supplement: Supplementary file 1 — Supporting Information [file ADVS-13-e14198-s001.docx]

**Uncoupling Metastasis and EMT in sgP19/kRAS-Driven Spontaneous Metastatic Liver Tumor Model**

Jingwen Wang^a^, Zijing Xu^a^, Lei Xu, Lishan Wang, Jiahao Geng, Xue Wang, Meng Xu, Daphne Superville, Melissa Reeves, Matthias Evert, Diego F. Calvisi^*^, Xin Chen^*^, Xinhua Song^*^

**Table of contents**

Supplementary Materials and Methods………………………………………… 1

References...…………………………………………………….………………… 6

Table S1……………………………………………………………………………. 7

Table S2……………………………………………………………………………. 8

Table S3……………………………………………………………………………..9

Table S4……………………………………………………………………………..10

Table S5……………………………………………………………………………..11

Sup. Fig 1……………………………………………………………………….......12

Sup. Fig 2…………………………………………………………………………...13

Sup. Fig 3…………………………………………………………………………...14

Sup. Fig 4…………………………………………………………………………...15

Sup. Fig 5…………………………………………………………………………...16

Sup. Fig 6…………………………………………………………………………...17

Sup. Fig 7…………………………………………………………………………...18

Sup. Fig 8…………………………………………………………………………...19

Sup. Fig 9…………………………………………………………………………...20

Sup. Fig 10………………………………………………………………………….21

Sup. Fig 11………………………………………………………………………….22

Sup. Fig 12………………………………………………………………………….23

Sup. Fig 13………………………………………………………………………….24

Sup. Fig 14………………………………………………………………………….25

Sup. Fig 15………………………………………………………………………….26

Sup. Fig 16………………………………………………………………………….27

**Supplementary Materials and Methods**

**Animals and Hydrodynamic Tail Vein Injection (HTVi)**

Wild-type *FVB/N* and *C57BL/6* mice were obtained from Charles River Laboratories (Wilmington, MA, USA). Stochastic multicolor Cre-reporter R26R-confetti homozygous mice (Confetti mice) were obtained from Jackson Laboratory (Sacramento, CA, USA). All the mice used for hydrodynamic injection were 6 to 8-week-old. *FVB/N*, *C57BL/6* or Confetti mice were received plasmid mixtures using hydrodynamic tail vein injection as described before ^[1]^. Briefly, the plasmids of various models are mixed according to a certain ratio (**Table S5**), then diluted into 0.9% saline (volume of saline equals 10% of mouse body weight), filtered, and injected into the lateral tail vein of seven weeks old mice in 7 to 9 seconds. Detailed plasmid mixture information used in the mouse studies is listed in **Table S5**. Mice were monitored by daily abdominal palpation and were euthanized when they developed a high burden of liver tumors, i.e., large abdominal masses. Mice were housed, fed, and monitored in accordance with protocols approved by the Committee for Animal Research at the University of California, San Francisco (San Francisco, CA)

***In vivo* lineage tracing**

Confetti mice were applied for lineage tracing procedures ^[2]^. Adeno associated virus 8 expressing hepatocyte specific Cre recombinase (AAV8-TBG-Cre) was injected into 4-week-old Confetti mice with 4×10^11^ genomic copies per ml (GC/ ml/ mice). Mice were injected with *sgP19/kRAS*/SB plasmid mixtures by HTVi and were sacrificed when moribund. Liver tissues, lymph node were collected and embedded in Tissue-Tek O.C.T compound (Sakura® Finetek, Cat# 4583). Frozen blocks were sectioned at 5 μm in thickness and were stained with anti-Vimentin primary antibody (1:50, Cell Signaling Technology, Cat#5741S). Nuclear DNA was stained with 300 nM DAPI (Millipore). Images were acquired using a Leica Sp5 AOBS confocal microscope (Mannheim, Germany) equipped with the following lenses: 203 (HCX PL FLUOTAR L NA0.40) dry objective; 103 (HCX PL APO CS NA0.40) dry objective; 403 (HCX PL APO NA0.85) dry objective; and a 633 (HCX PL APO NA1.30) glycerol objective. Scans were performed in series for XFP excitations: for RFP, a red diode laser emitting at 561 nm was applied, for spectrally nearly indistinguishable GFP and YFP, the argon laser 488 nm line was used; and blue CFP was excited using a laser line at 458 nm. Vimentin was obtained while using 684 nm laser and DAPI was obtained with 405 nm laser. The acquired images were processed with Photoshop.

**Cell culture and in vitro studies**

KMCH iCCA cell line used for the in vitro studies was a generous gift by Dr. Gregory J. Gores (Mayo Clinic, Rochester, MN). Cell lines were validated (Genetica DNA Laboratories, Burlington, NC, USA) and maintained as monolayer cultures in Dulbecco’s modified Eagle medium with 10% fetal bovine serum (FBS; Gibco, Grand Island, NY, USA), 100 U/mL penicillin, and 100 g/mL streptomycin (Gibco). Transfection with lentivirus assay were performed as described before in detail ^[3]^. In brief, for transfection of EGFP or TGFβ1, pCW57.1-EGFP or pCW57.1-TGFβ1 lentivirus was added to the culture medium when cells reached ~60% confluency in 60 × 15 mm culture dishes. 72 hours later, cells were passaged in 100 × 20 mm dishes with culture medium containing puromycin at the concentration of 5 μg/ml. At least 3 days of selection, EGFP and TGFβ1 expression were induced by Doxycycline at a concentration of 4 μg/ml.

**Quantitative real-time reverse transcriptase polymerase chain reaction (qRT-PCR)**

Total mRNA from cells and liver tissues was extracted using the Quick RNA Miniprep kit (Zymo Research, Irvine, CA, USA). mRNA expression of target genes was detected by quantitative real-time polymerase chain reaction (qRT-PCR) using SYBR Green Master Mix (Applied Biosystems, Foster City, CA, USA) in a QuantStudio™ 6 Flex system (Applied Biosystems). 18S rRNA was used to normalize the expression of each gene. Cycling conditions were as follows: denaturation at 95°C for 10 min, 35 cycles at 95°C for 15 s, 60°C for 1 minute, 72°C for 30 seconds. Primers used in the experiment are listed in **Table S4**. Quantitative values for each gene were calculated by using the PE Biosystems Analysis software and expressed as number target (NT). N^−∆Ct^, wherein ∆Ct value of each sample was calculated by subtracting the average Ct value of the target gene from the average Ct value of the *GAPDH* gene.

**Western blot analysis**

iCCA cell lines and liver tissues were homogenized in the M-PER mammalian protein extraction reagent (Cat. N. 78501; Thermo Fisher Scientific, Rockford, IL) with 1x Complete Protease Inhibitor Cocktail (ThermoFisher [Scientific](http://www.thermoscientific.com/en/product/ki-67-rabbit-monoclonal-antibody.html), [Waltham,](https://www.google.com/search?es_sm=122&q=waltham+ma&stick=H4sIAAAAAAAAAOPgE-LSz9U3MCooMTBJU-IAsTOqjE21tLKTrfTzi9IT8zKrEksy8_NQOFYZqYkphaWJRSWpRcUAAxikqkQAAAA&sa=X&ved=0CI4BEJsTKAEwFGoVChMI2tX76cXoyAIVTe1jCh2YUABw) MA). Bio-Rad Protein Assay Kit (Bio-Rad, Hercules, CA) was used to detect the protein concentrations. Protein lysates were denatured by boiling in Tris–Glycine SDS Sample Buffer (Invitrogen, Grand Island, NY). 30 μg proteins were separated by SDS-PAGE gel and transfected onto nitrocellulose membranes (Invitrogen). Membranes were blocked in 10% non-fat dry milk in Tris-buffered saline containing 0.1% Tween 20 for 1-2 h and incubated with specific primary antibodies for 2 h or overnight (**Table S2**). Subsequently, a horseradish peroxidase-secondary antibody diluted 1:5000 for 1 h was applied and was revealed with the Super Signal West Pico Chemiluminescent Substrate (Pierce Chemical Co., New York, NY).

**Histology, Immunohistochemistry (IHC), and Immunofluorescence (IF) analysis**

Liver samples were fixed for at least 24 hours in Zinc Formal-Fix (Thermo Shandon Ltd, Runcorn, United Kingdom) and stored at 4 °C prior to paraffin embedding. Sections of 5 μm in thickness were used for subsequent assay. For IHC of HNF-4α, Tris/EDTA buffer (pH 9.0) was used as antigen retrieval buffer, for Cleaved-Caspase-3 IHC staining, antigen retrieving was done in EDTA buffer (pH 8.0), and sodium citrate buffer (pH 6.0) was performed for all other targets. Slides emerged in retrieval buffer and were heated in a microwave on high level for 10 minutes. After cooling down, slices were blocked by goat serum and Avidin-Biotin blocking kit (Vector Laboratories, Burlingame, CA). Specimens were incubated with designated primary antibody at 4 °C overnight. Detailed information for antibodies used are listed in **Table S3**. Subsequently, 3% hydrogen peroxide was applied for 10 min to quench endogenous peroxidase activity. Slices were incubated in the biotin conjugated secondary antibody (Life Technology, Waltham, MA) at a 1:500 dilution for 30 min at room temperature. The Vectastain Elite ABC Kit (Vector Laboratories) and DAB substrates (Dako North America, Carpinteria, CA) were applied to visualize the immunostainings. Vimentin and CK19 staining were quantified using the Image J software (NIH; <https://imagej.nih.gov/ij/>).

For IF, after antigen retrieval block slides in 10% serum for 1 hour, sections were incubated with primary antibodies overnight at 4°C. Subsequently, secondary antibodies were applied for 1 hour at room temperature and nuclear DNA was stained with 300 ηM DAPI (Millipore). IHC and IF antibodies used in the experiment are listed in **Table S3**.

**TIDE analysis**

The DNeasy Blood & Tissue Kit (Cat. N. 69504; QIAGEN, Germany) was used to extract DNA from normal mice liver and *sgP19/kRAS* model mice liver tumor tissues with the *FVB/N* background. The amplification conditions were: 95 °C for 5 minutes, followed by 35 cycles of 95 °C for 30 s, 55 °C for 30 s, and 72 °C for 30 s; followed by 72 °C for 5 minutes. Primers were designed to specifically amplify *P19* and the primer sequence was listed in **Table S4**. Sanger sequencing of the samples was performed by GENEWIZ (US). The sequencing results of the two groups and the sgRNA sequence (CACTGTGAggattcagcgcg) were input into the TIDE website (<http://tide.nki.nl/>) to evaluate the mutation status of *P19*.

**Reference**

1. Chen X, Calvisi DF. Hydrodynamic transfection for generation of novel mouse models for liver cancer research. The American journal of pathology 2014; 184:912-23.
2. Reeves MQ, Kandyba E, Harris S, Del Rosario R, Balmain A. Multicolour lineage tracing reveals clonal dynamics of squamous carcinoma evolution from initiation to metastasis. Nature cell biology 2018; 20:699-709.
3. Zhang S, Song X, Cao D, et al. Pan-mTOR inhibitor MLN0128 is effective against intrahepatic cholangiocarcinoma in mice. Journal of hepatology 2017; 67:1194-203.

**Supplementary Tables**

**Supplementary Table 1** Summary of metastasis-related phenotypes

|  |  | **Percentage of metastasis (%)** | | | |
| --- | --- | --- | --- | --- | --- |
| Oncogenes | Type(n=10) | Lymph  node | Lung | Kidney | Uterus |
| *sgP19/kRAS* | *C57BL/6J* | ~100(n=10) | ~50(n=5) | ~30(n=3) | ~10(n=1) |
| *sgP19/kRAS* | *FVB/N* | ~100(n=10) | ~50(n=5) | ~40(n=4) | ~10(n=1) |

The rate of Metastasis in different organs

| Organs | Phenotype | Percentage (%) |
| --- | --- | --- |
| Lymph node | iCCA | 70 |
|  | Sarcomatoid | 30 |
| Lung | iCCA | 90 |
|  | Sarcomatoid | 10 |
| Kidney | iCCA | ~5 |
|  | Sarcomatoid | ~95 |

Tumor phenotype in different organs

**Supplementary Table 2:** List of antibodies used for Western blot analysis

| WB | Catalogue No. | Company | Dilution |
| --- | --- | --- | --- |
| CK19 | Ab133496 | Abcam | 1:2000 |
| E-cadherin | 610181 | BD Biosciences | 1:2000 |
| GAPDH | 5174 | Cell Signaling Technology | 1:10000 |
| HA-tag | 3724 | Abcam | 1:1000 |
| p-SMAD2/3 | 8824 | Cell Signaling Technology | 1:500 |
| Vimentin | 5741 | Cell Signaling Technology | 1:1000 |
| SMAD2/3 | ab202445 | Abcam | 1:1000 |
| TGFBR1 | bs-0638R | Bioss | 1:1000 |
| TGFBR2 | 27212-1-AP | Proteintech | 1:500 |
| kRAS | 12063-1-AP | Proteintech | 1:5000 |
| ZEB1 | 13619 | Cell Signaling Technology | 1:1000 |
| 2^nd^ Antibodies | Concentration | Company | Catalogue No. |
| Goat anti-Mouse | 1:500 | Invitrogen | A11001 |
| Goat anti-Rabbit | 1:500 | Invitrogen | B2770 |

**Supplementary Table 3:** Antibodies Used for IHC and IF

| **IHC** | | | Catalogue No. | Company | Dilution |
| --- | --- | --- | --- | --- | --- |
| **CK19** | | | Ab133496 | Abcam | 1:1000 |
| **KI67** | | | RM-9106-S1 | Cell Signaling Technology | 1:150 |
| **Vimentin** |  | | 5741 | Cell Signaling Technology | 1:50 |
| **TGFBR1** |  | | Ab215715 | Abcam | 1:500 |
| **TGFBR2** |  | | PA5-88257 | Thermo Fisher Scientific | 1:50 |
| **CK7** |  | | Ab181598 | Abcam | 1:8000 |
| **S100A4** |  | | ab197896 | Abcam | 1:2000 |
| **α-SMA** |  | | 19245S | Cell Signaling Technology | 1:500 |
| **ZEB1** | | | 13619 | Cell Signaling Technology | 1:100 |
| **IF** | | | Concentration | Company | Catalogue No. |
| **Vimentin** | | | 1:50 | Cell Signaling Technology | 14074 |
| **CK19** | | | 1:3000 | Abcam | Ab133496 |
| **Ki67** | | | 1:2000 | Servicebio | GB111141-100 |
| **2^nd^ Antibodies** | | Concentration | | Company | Catalogue No. |
| **Goat anti-Rabbit** | | | 1:500 | Invitrogen | **Q-11471MP** |

**Supplementary Table 4:** The primer sequences used in the article

| Mouse | Sense 5'-3' | Antisense 5'-3' |
| --- | --- | --- |
| Twist1 | CGGCCAGGTACATCGACTTC | GCTCGTGGGCCACATAGC |
| Slug | GCTGCAAGTACTGTGACAAGGAA | TGTGGGTTCGAATGTGCATCT |
| Zeb1 | TGATGAAAACGGAACACCAGA | GTTGTCCTCGTTCTTCTCATG |
| Snail1 | CCATGTCTGGACCTGGTTCCT | GGGTACAAAGGCACTCCATCA |
| P19 | AAAGTTAACCGGAGCGAAAGC | CTTCGGAGGGCCTTTCCTAC |
| 18S rRNA | CGGCTACCACATCCAAGGAA | GCTGGAATTACCGCGGCT |

**Supplementary Table 5:** Plasmid ratios for all models and the number of animals in each group

| Figure | Group | Plasmid mixture |
| --- | --- | --- |
| Fig. 1,3 | Experiment(n=10) | 10 μg *sgP19*+25 μg *kRAS*+7 μg *SB* |
| Fig. 4 | Control(n=4) | 10 μg *sgP19*+25 μg *kRAS*+40 μg *PT3*+7 μg *SB* |
|  | Experiment(n=5) | 10 μg *sgP19*+25 μg *kRAS*+40 μg *TGFβ1*+7 μg *SB* |
| Supplementary. Fig. 9 | Control(n=5) | 10 μg *sgP19*+25 μg *kRAS*+40 μg *PT3*+7 μg *SB* |
|  | Experiment(n=5) | 10 μg *sgP19*+25 μg *kRAS*+40 μg *Snail1*+7 μg *SB* |
| Supplementary. Fig. 9 | Control(n=5) | 10 μg *sgP19*+25 μg *kRAS*+40 μg *PT3*+7 μg *SB* |
|  | Experiment(n=5) | 10 μg *sgP19*+25 μg *kRAS*+40 μg *Twist1*+7 μg *SB* |
| Fig. 5 | Control(n=5) | 10 μg *sgP19*+25 μg *kRAS*+40 μg *PT3*+7 μg *SB* |
|  | Experiment(n=6) | 10 μg *sgP19*+25 μg *kRAS*+40 μg *Zeb1*+7 μg *SB* |
| Fig.6  Supplementary. Fig. 13 | Control(n=6) | 10 μg *sgP19*+25 μg *kRAS*+40 μg *sgGFP* +7 μg *SB* |
|  | Experiment(n=10) | 10 μg *sgP19*+25 μg *kRAS*+40 μg *sgZeb1* +7 μg *SB* |
| Fig. 7  Supplementary. Fig. 14 | Control(n=6) | 10 μg *sgP19*+25 μg *kRAS*+40 μg *sgGFP* +7 μg *SB* |
|  | Experiment(n=6) | 10 μg *sgP19*+25 μg *kRAS*+40 μg *sgTGFbr1&2* +7 μg *SB* |
| Supplementary. Fig. 16 | Control(n=6) | 10 μg *sgP19*+25 μg *kRAS*+40 μg *PT3*+7 μg *SB* |
|  | Experiment(n=7) | 10 μg *sgP19*+25 μg *kRAS*+40 μg *Smad7*+7 μg *SB* |

**Supplementary Figures and Figure legends**


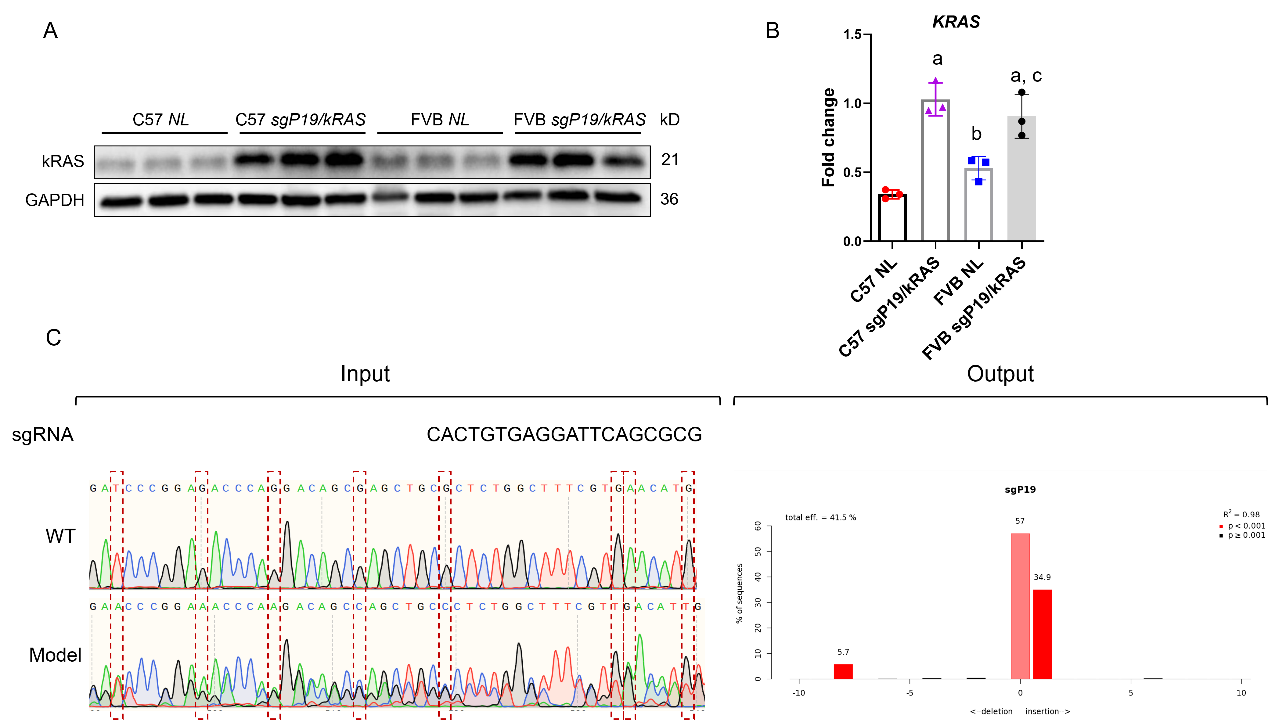


**Supplementary Figure 1:** (A) Western blot results showing *kRAS* expression of *C57BL/6* and *FVB/N* normal liver, *sgP19/kRAS* *C57BL/6*, and *FVB/N* tumor tissues. GAPDH was used as a loading control. (B) Quantification results of *kRAS*. (C) The mutation result of *P19* in the wild type and *sgP19/kRAS* model with *FVB/N* background was detected through TIDE. Tukey–Kramer test: at least *P < 0.05*; a, vs C57BL/6 NL; b, vs C57BL/6 *sgP19/kRAS* mice; c, vs FVB/N NL.


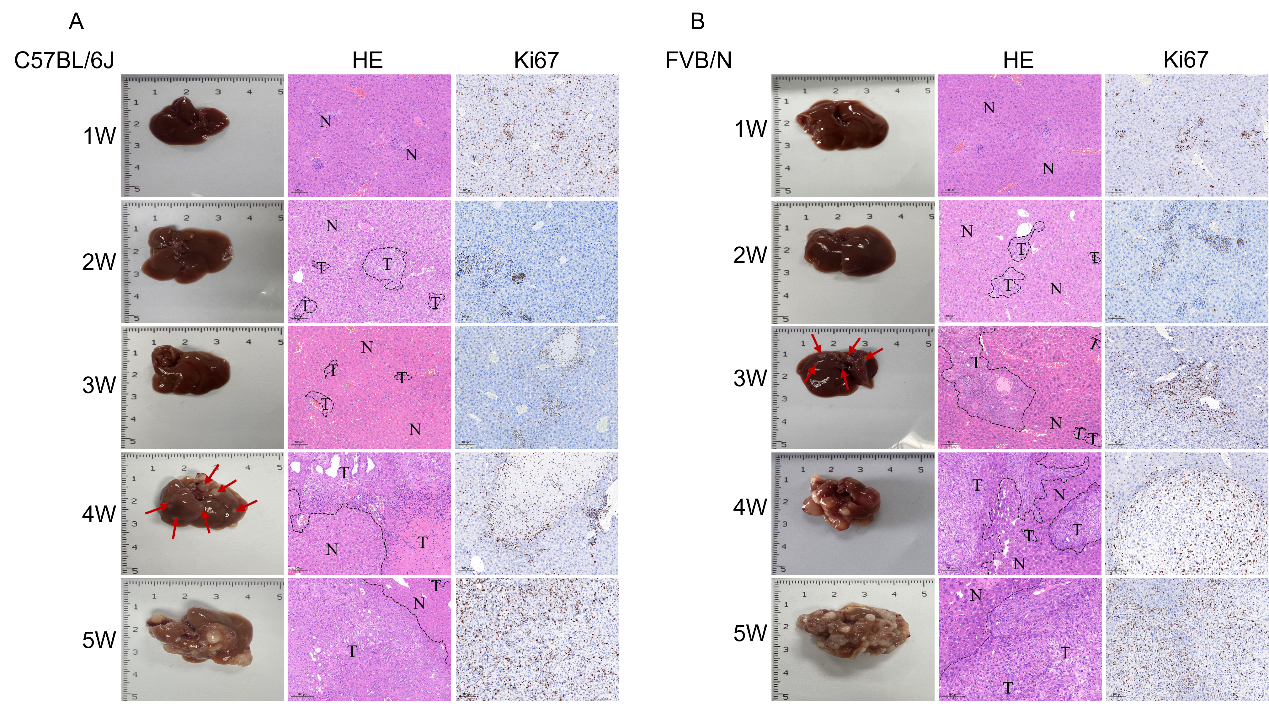


**Supplementary Figure 2:** Representative images of H&E and Ki67 staining of mouse livers in the *sgP19/kRAS* models with *C57BL/6* (A) and *FVB/N* (B) backgrounds are presented in chronological order. Scale bar: 100 μm. Abbreviation: T, Tumor; N, normal liver.


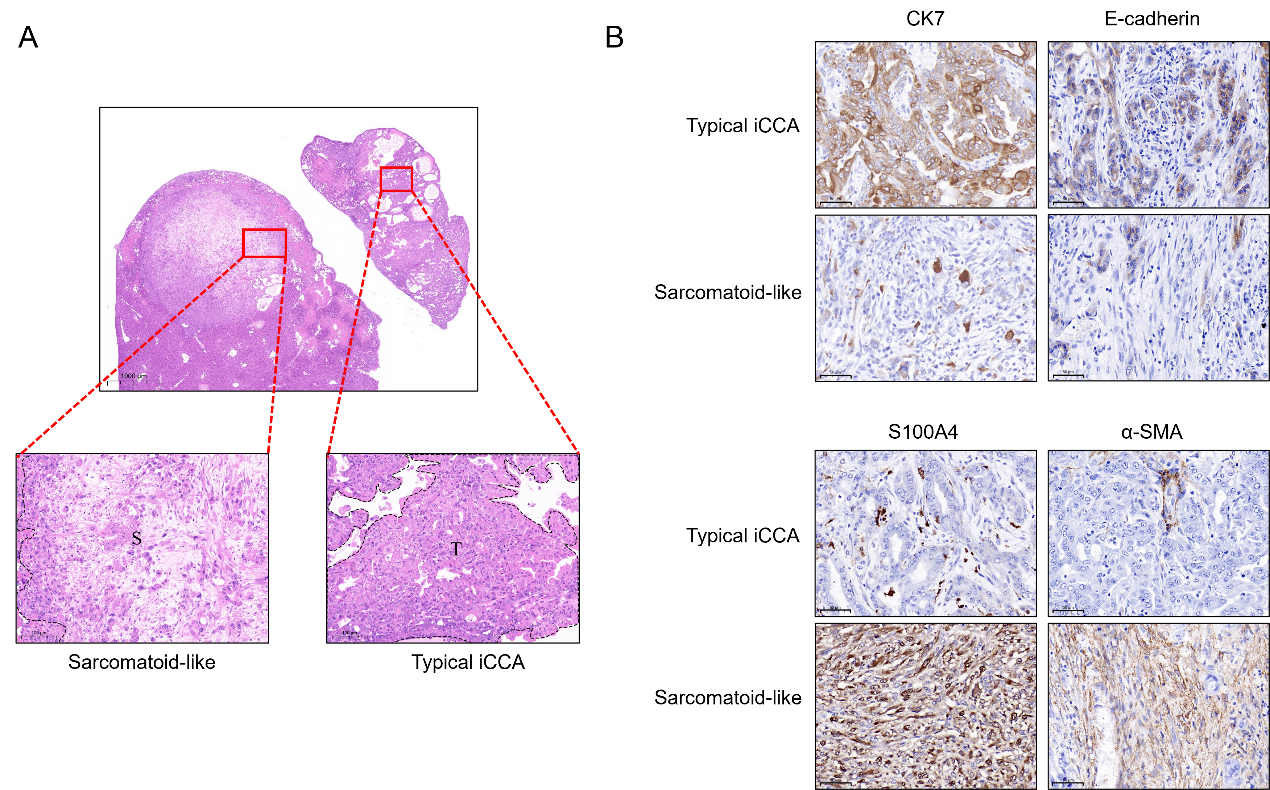


**Supplementary Figure 3:** EMT is induced in the *sgP19/kRAS* mixed iCCA model. Representative images of CK7, S100A4, E-cadherin and α-SMA staining in *sgP19/kRAS* mouse liver tissues. Scale bar: 1000 μm; Higher magnification, 100 μm (A); 50 μm (B). Abbreviation: T, Typical iCCA; S, Sarcomatoid-like.


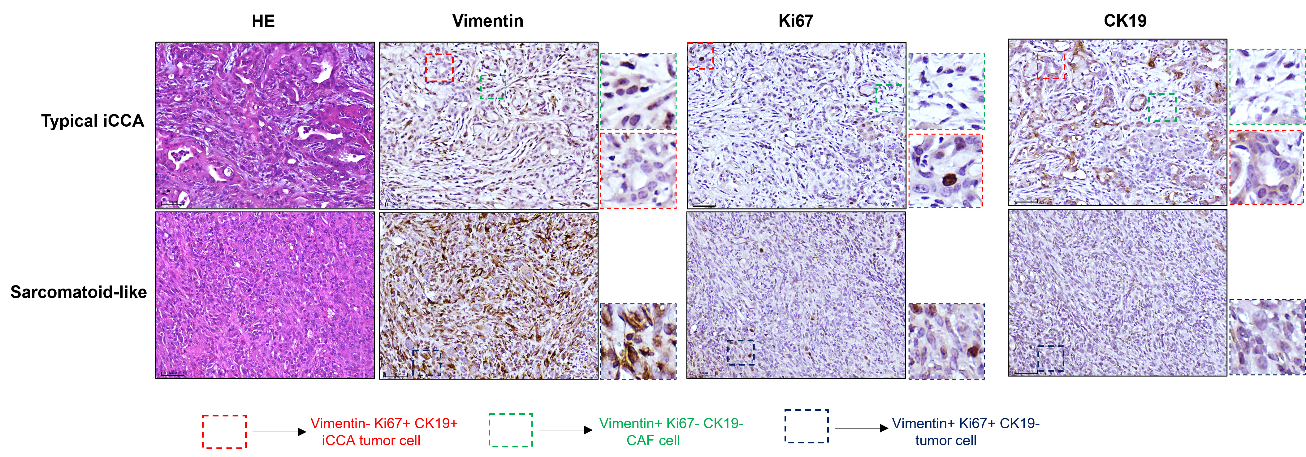


**Supplementary Figure 4:** IHC analysis of Vimentin (+) cells in the *sgP19/kRAS* mixed iCCA model. IHC staining of Vimentin, Ki67 and CK19 was performed on the consecutive section, and representative images are presented. In typical iCCA lesions, CK19 (+) Vimentin (-) Ki67 (+) cells represent iCCA cells. Vimentin (+) CK19 (-) Ki67 (-) cells are CAFs. In Sarcomatoid-like lesions, CK19 (-) Vimentin (+) Ki67 (+) cells represent tumor cells. These tumor cells were derived from hepatocytes and underwent EMT. Scale bar: 50 μm.


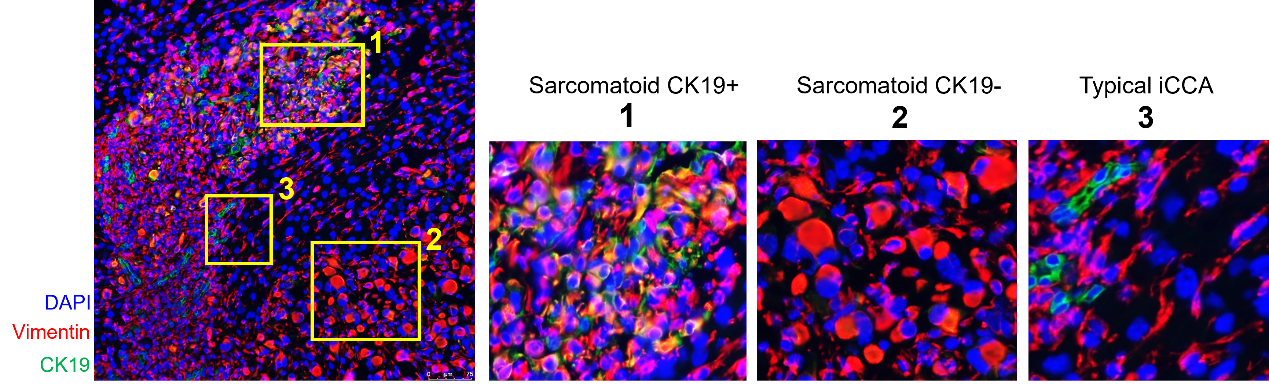


**Supplementary Figure 5:** Expression of Vimentin and CK19 levels in *sgP19/kRAS* mouse liver tissues. Representative immunofluorescence staining images of Vimentin and CK19 in *sgP19/kRAS* mouse liver tissues. Scale bar, 75 μm.


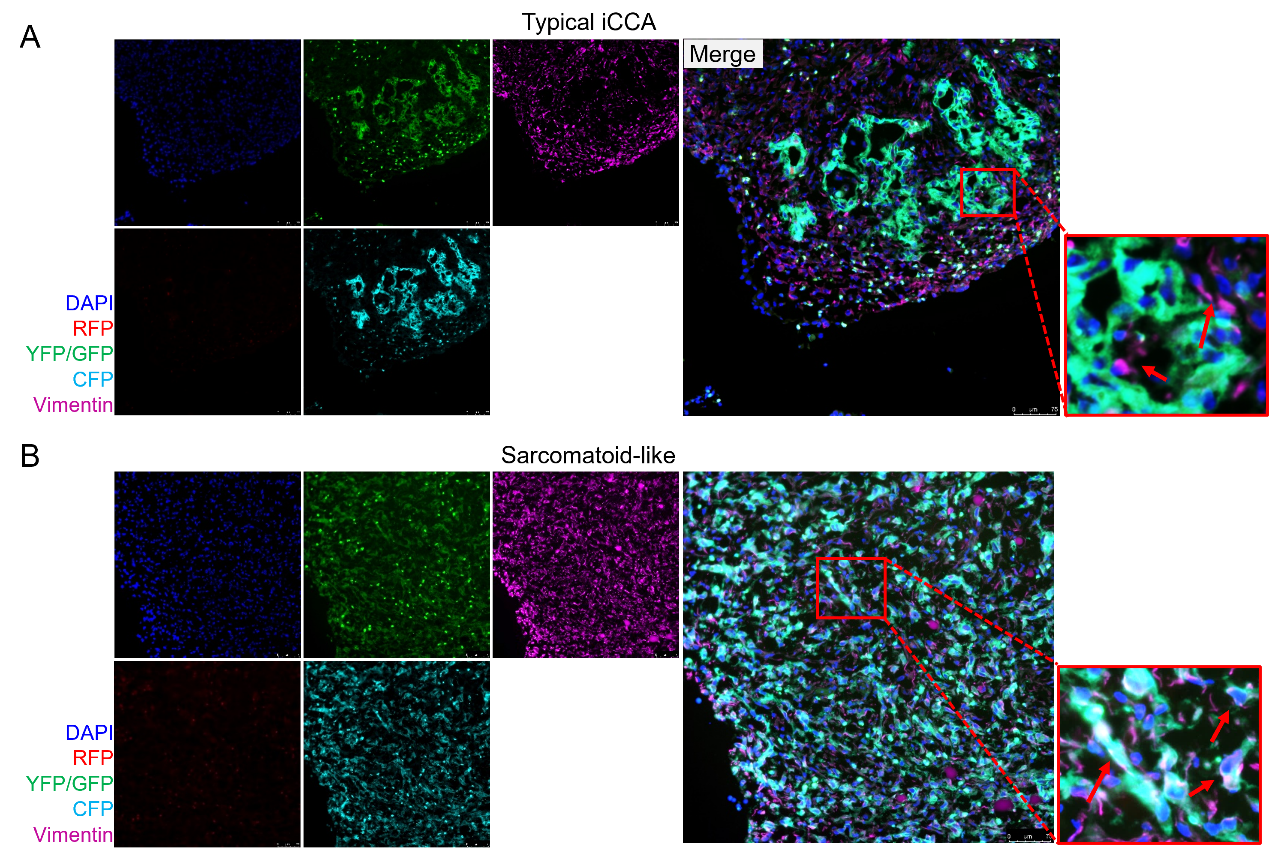


**Supplementary Figure 6:** Immunofluorescence staining of Vimentin in *sgP19/kRAS* confetti mouse lymph node metastasis tissues. Scale bar: 75 μm.


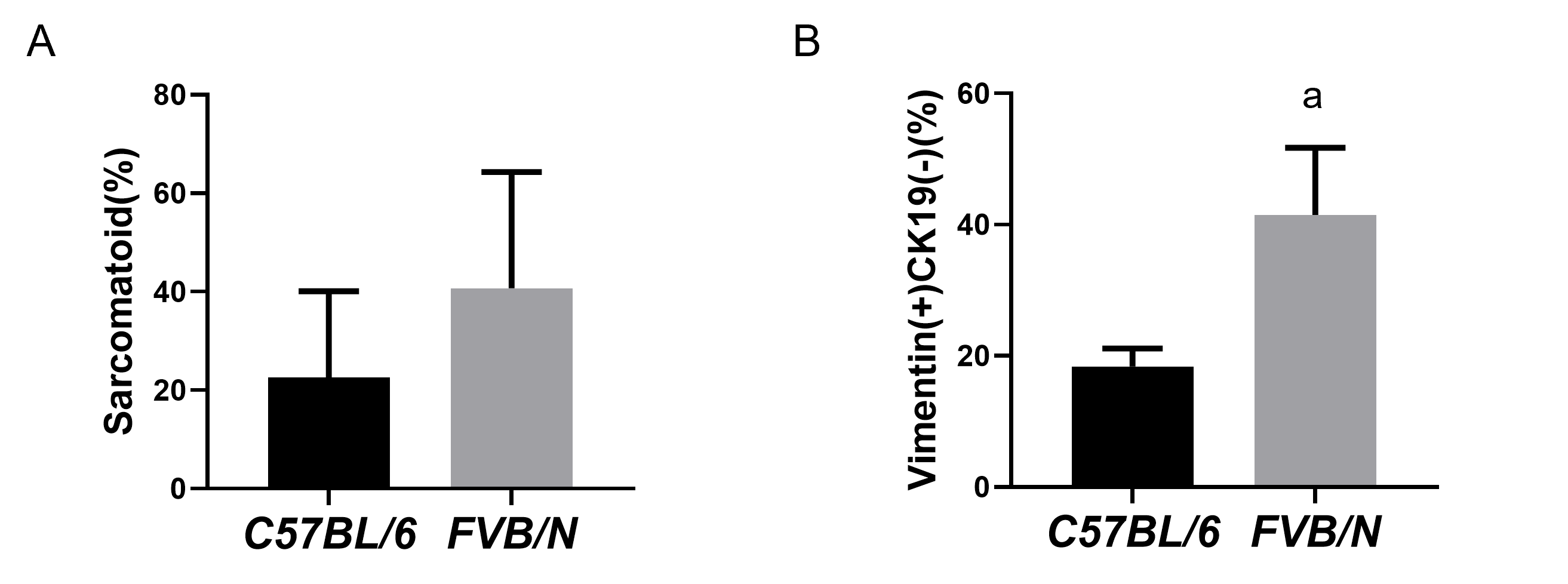


**Supplementary Figure 7:** EMT progresses more rapidly in mice with the *FVB/N* background. Statistical results of sarcomatoid lesions (A) and Vimentin (+) CK19 (-) tumor cells (B) in mice with *FVB/N* and *C57BL/6* backgrounds. Tukey–Kramer test: at least *P < 0.05*; a, vs *C57BL/6*.


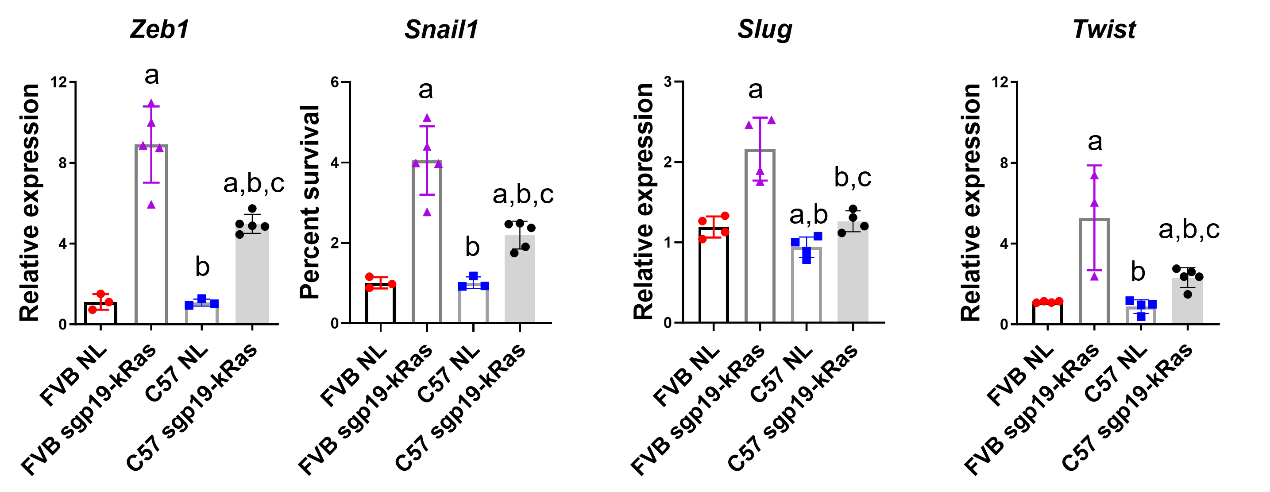


**Supplementary Figure 8:** EMT-activating transcriptional factors *Zeb1, SnaiI1, Slug* and *Twist1* at transcriptional level was assessed by qPCR. Data were analyzed and normalized using the −ΔΔCt method and presented as mean ± SD; Tukey–Kramer test: at least *P < 0.05*; a, vs FVB/N NL; b, vs FVB/N *sgP19/kRAS* mice; c, vs *C57BL/6* NL. Abbreviation: NL, normal liver.


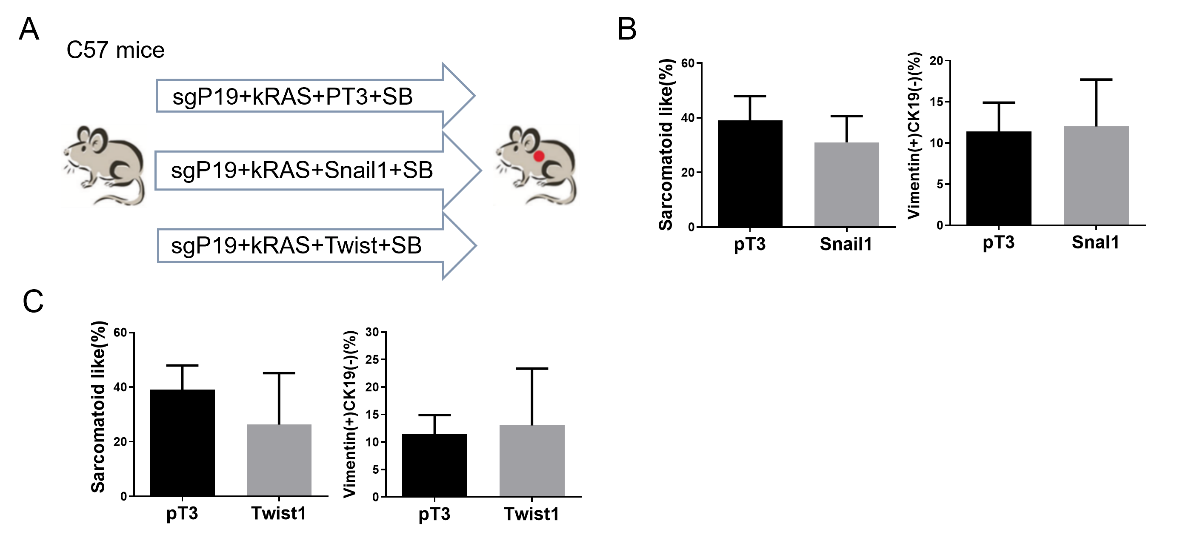


**Supplementary Figure 9:** Overexpression of *Snail1* or *Twist* in *sgp19-kRAS* iCCA model of *C57BL/6* mice. (A) Study design. *C57BL/6* mice were injected with *sgp19/kRAS/PT3*, *sgp19/kRAS/snail1*, or *sgp19/kRAS/twist1*, plasmids, respectively. Mice were monitored and sacrificed when moribund. (B) Percentage of sarcomatoid-like and Vimentin+CK19- staining tumor lesions of *sgp19/kRAS/PT3* (n=5), *sgp19/kRAS/snail1* (n=5) or *sgp19/kRAS/twist1* (n=5) mice.


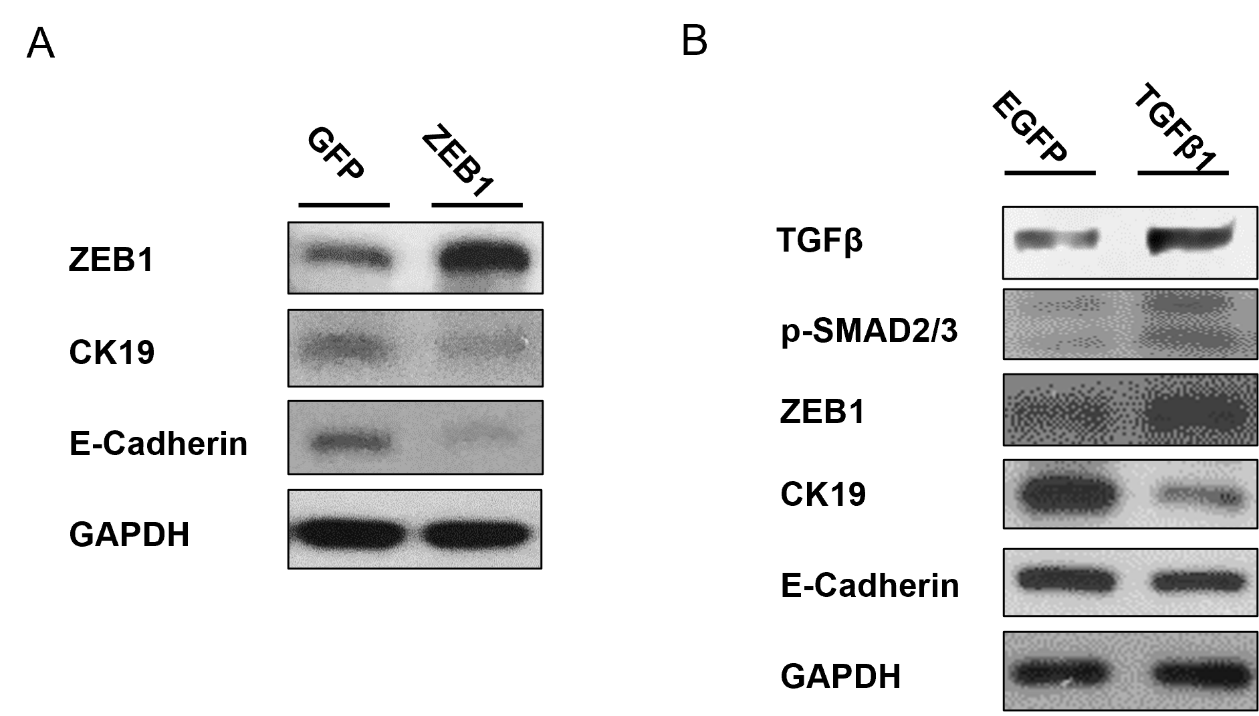


**Supplementary Figure 10:** *TGFβ1* upregulated *ZEB1* to promote EMT in ICC cell line. (A) Western blot analysis of relative protein expression in KMCH ICC cell line transfected with control GFP or Zeb1. (B) Western blot analysis of relative protein expression in KMCH ICC cell line transfected with control EGFP or *Tgfβ1*. GAPDH was used as a loading control.


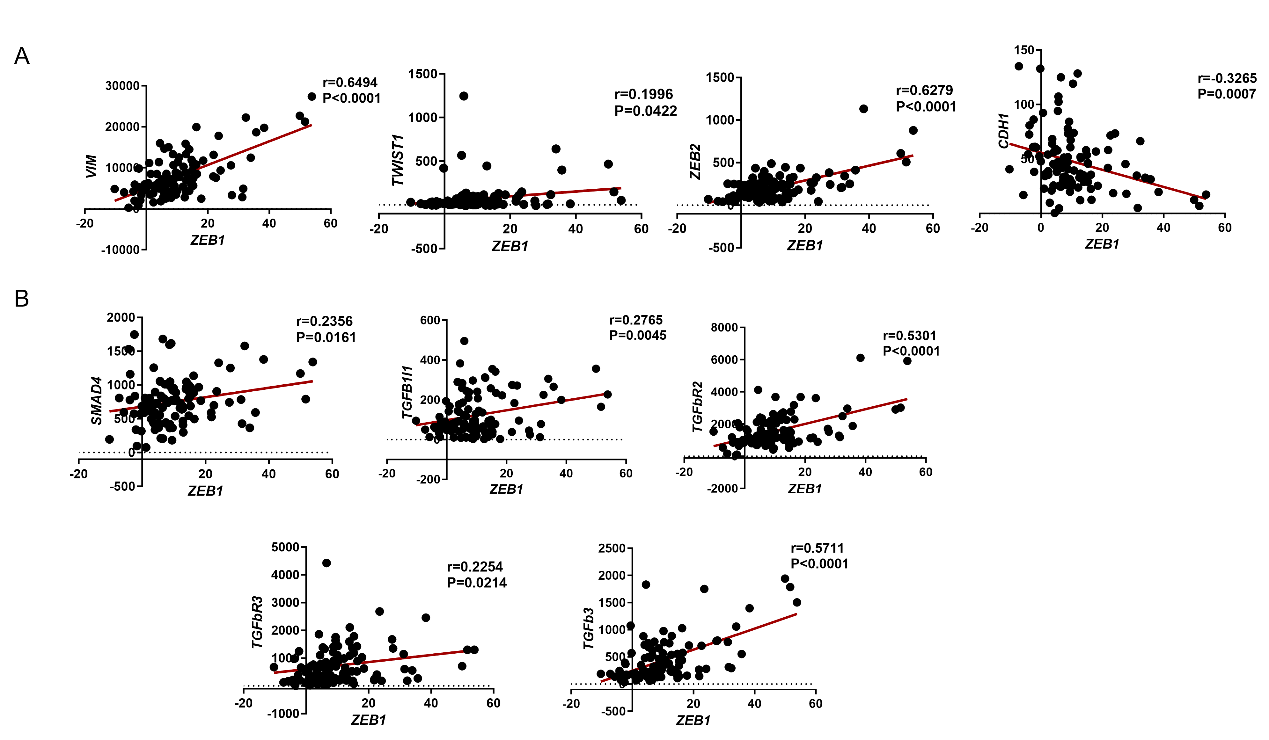


**Supplementary Figure 11:** Correlation analysis of *ZEB1* expression with EMT (A) and TGFβ pathway related genes (B) in human iCCA samples (n=104) from the National Cancer Institute (NCI) database. The scatter plot displays individual sample points. The red line represents a simple linear regression fit, indicating the trend of the relationship between the two gene expression levels. The slope of the line suggests the degree of correlation, with statistical significance assessed by the correlation coefficient (r) and the P-value.


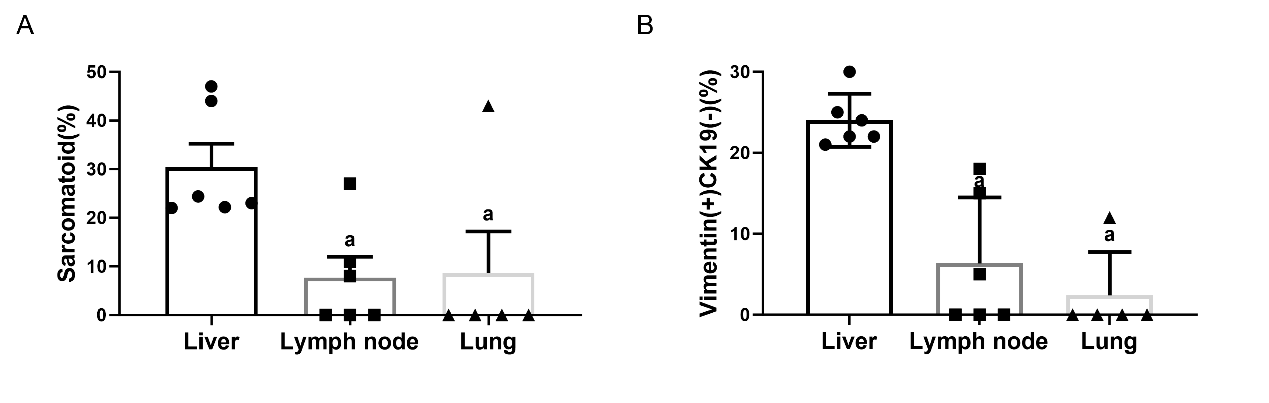


**Supplementary Figure 12:** The percentages of sarcomatoid tumor lesions (A) and Vimentin (+) CK19 (-) tumors (B) in the liver, lymph node and lung of *sgP19/kRAS* iCCA mixed model. Tukey–Kramer test: *P < 0.001*; a, vs. Liver; b, vs. Lymph node.


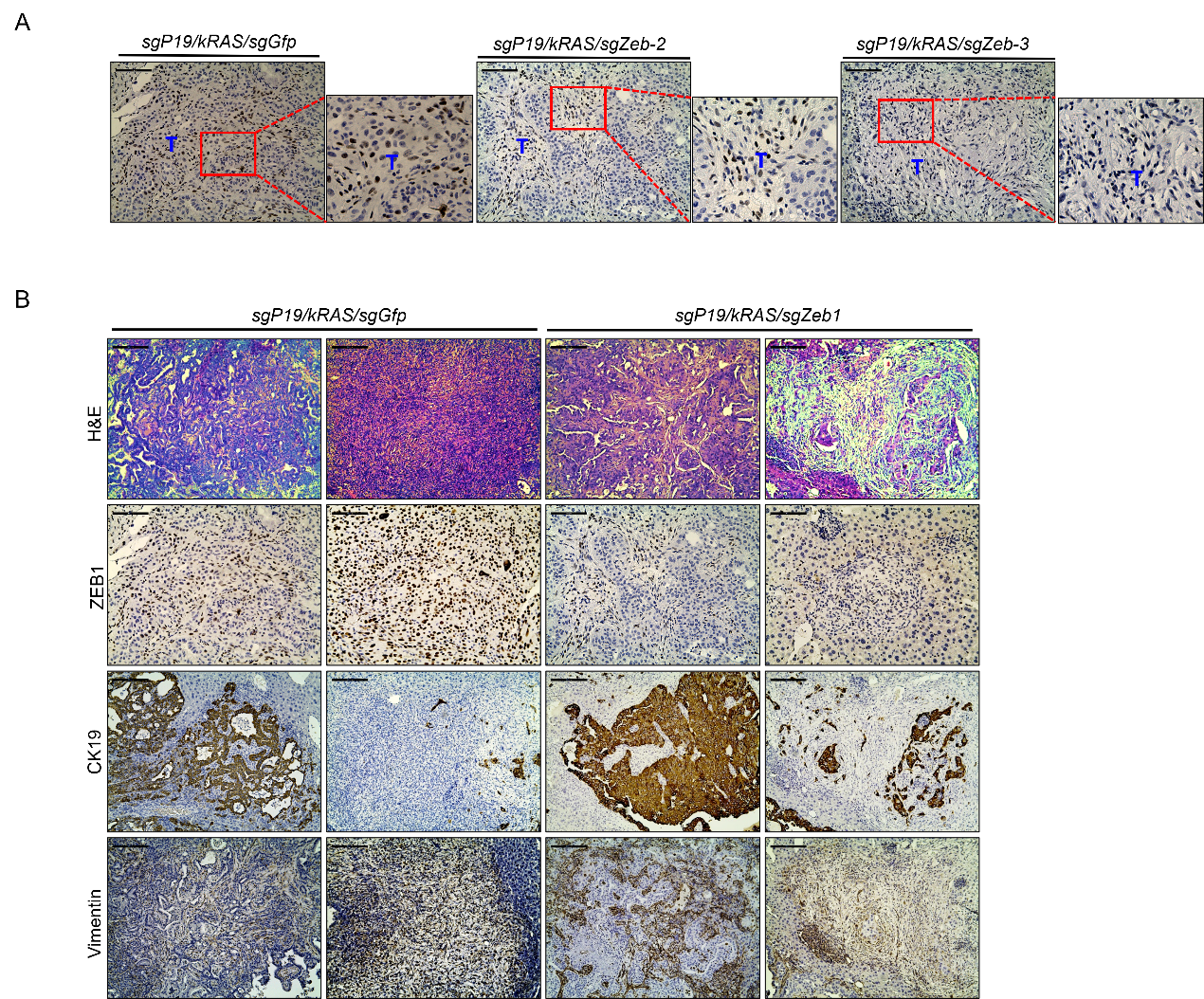


**Supplementary Figure 13:** (A) Zeb1 staining in *sgP19/kRAS/sgGfp* and three sgZeb1 constructs (*sgP19/kRAS/sgZeb1)* tumor tissues. (B) Representative images of hematoxylin and eosin (H&E), ZEB1, CK19, and Vimentin staining in *sgP19/kRAS/sgGfp* (n=6) and *sgP19/kRAS/sgZeb1* (n=10) mouse liver tissues. Original magnification: 200x; scale bar: 100 µm. Abbreviation: H&E, hematoxylin and eosin staining.


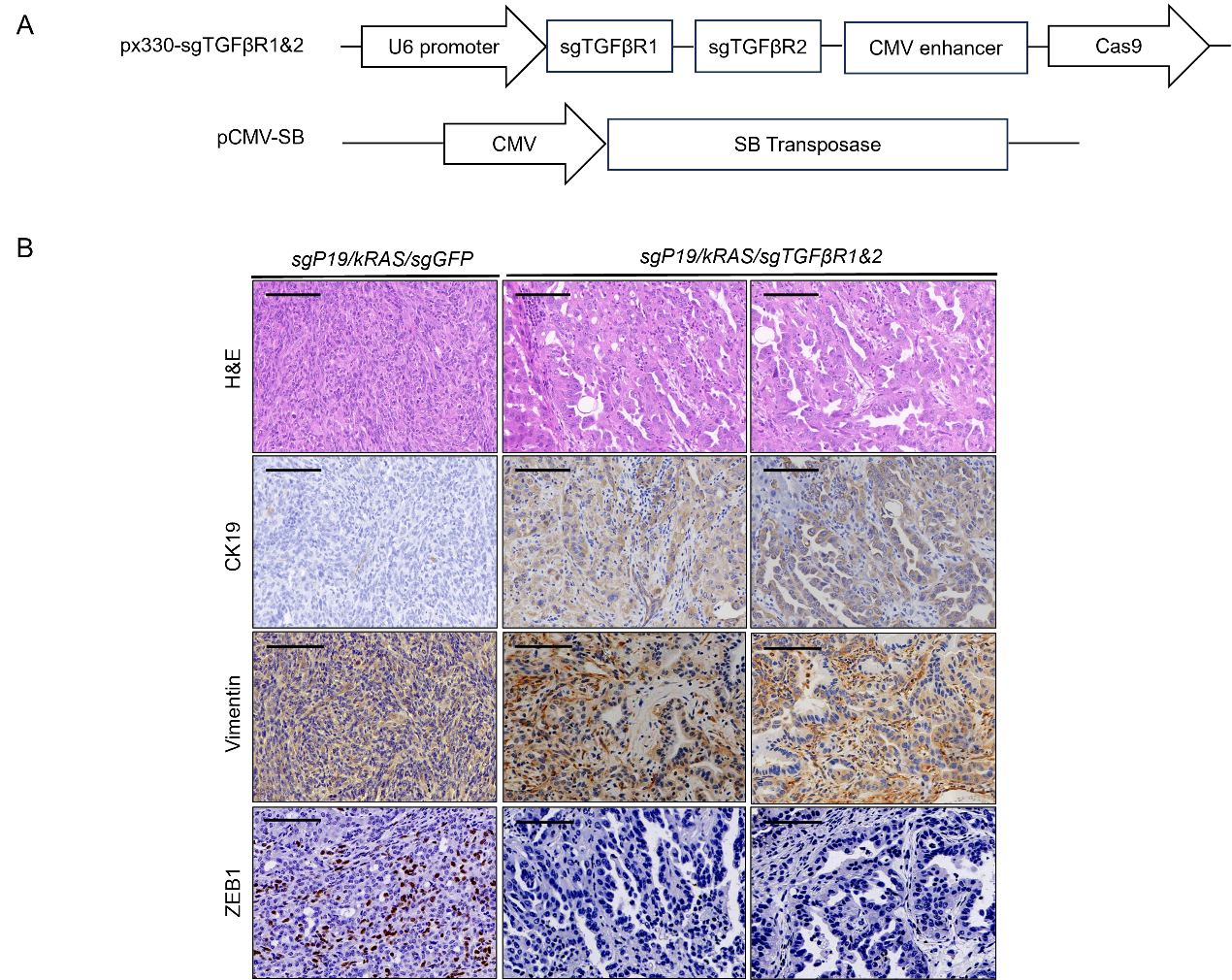


**Supplementary Figure 14:** (A) Schematic of vectors injected into mice. (B) Representative images of hematoxylin and eosin (H&E), CK19, Vimentin and ZEB1 staining in *sgP19/kRAS/sgGfp* (n=6) and *sgP19/kRAS/sgTgfbr1&2* (n=6) mouse liver tissues. Original magnification: 200x; scale bar: 100 µm.


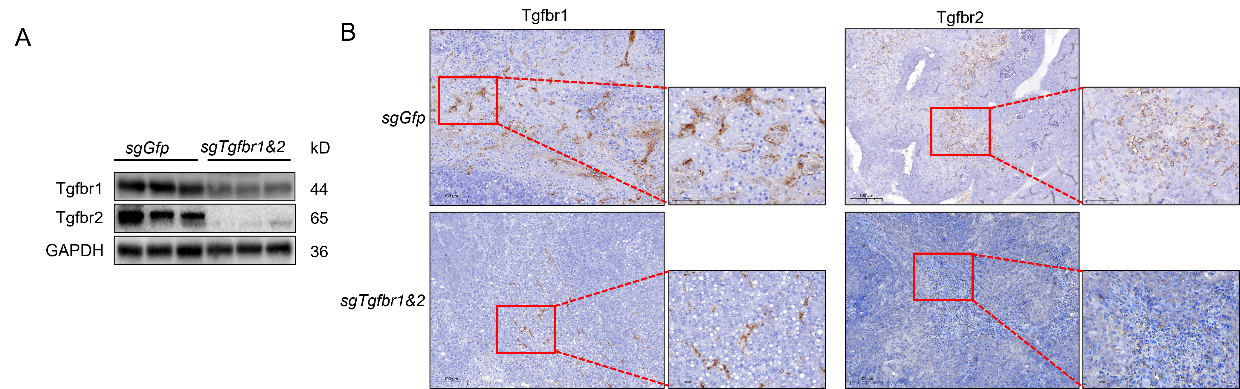


**S****upplementary Figure 15:** (A) Western blot and (B) immunohistochemistry results showing *Tgfbr1* and *Tgfbr2* expression in the *sgP19/kRAS/sgTgfbr1&2* and *sgP19/kRAS/sgGFP* models. GAPDH was used as a loading control. scale bar: 100 µm; Higher magnification, 50 μm.


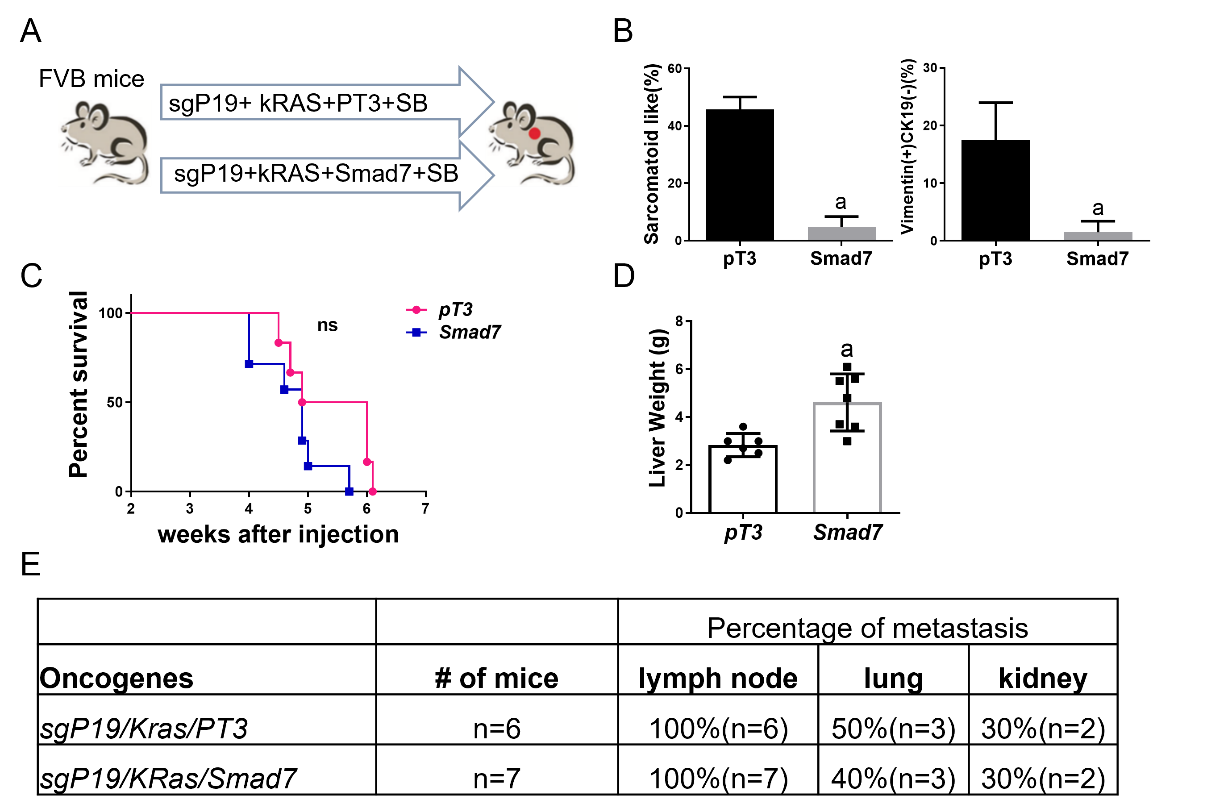


**Supplementary Figure 16:** Inactive TGFβ inhibits EMT without delaying carcinogenesis or suppressing metastasis in *sgP19/kRAS*-induced iCCA mice model. (A) Study design. FVB/N mice were injected with *sgP19/kRAS/PT3* or *sgP19/kRAS/Smad7* plasmids, respectively. Mice were monitored and sacrificed when moribund. (B) Percentage of sarcomatoid and Vimentin (+) CK19 (-) staining tumor lesions. (C) Survival curve showing that *ZEB1* knockout does not prolong *sgP19/kRAS* mouse survival. (D) Liver weight of FVB mice injected with *sgP19/kRAS/PT3* (n=6) or *sgP19/kRAS/Smad7* (n=7). (E) Summary of the percentage of metastasis in lumph node, lung and kidney of *sgP19/kRAS/PT3* or *sgP19/kRAS/Smad7* mice. Student’s t test: at least *P < 0.05*; a, vs pT3; *sgP19/kRAS/PT3* mice.
